# Seven-Coordinate Lanthanide Bis-Halide Bis-Tetrathiometallate Complexes: A Compelling Platform for Luminescent and Magnetic Properties

**DOI:** 10.1021/acs.inorgchem.5c04468

**Published:** 2025-12-16

**Authors:** Marie A. Perrin, Salauat R. Kiraev, Julia Specht, Liam Grunwald, Fabrice Pointillart, Olivier Cador, Boris Le Guennic, Olivier Maury, Victor Mougel

**Affiliations:** † Department of Chemistry and Applied Biosciences, 27219 ETH Zurich, Vladimir-Prelog-Weg 1-5/10, 8049 Zurich, Switzerland; ‡ CNRS, LCH (Laboratoire de Chimie) UMR 5182, ENS de Lyon, 69342 07 Lyon Cedex, France; § Université de Rennes, CNRS, ISCR (Institut des Sciences Chimiques de Rennes) - UMR 6226, 35000 Rennes, France

## Abstract

To harness the unique luminescent and magnetic properties of lanthanides, precise control over their coordination sphere is essential, whether to minimize deactivation processes or maximize magnetic anisotropy. The traditional approaches rely on the use of large organic ligands bearing strong atom donors to enforce low coordination numbers of Ln ions. Herein we explored an alternative approach, constraining low coordination numbers via a better control of the primary coordination sphere of the Ln center using fully inorganic sulfur-based ligands, tetrathiotungstates. This strategy led to the preparation of an isostructural series of 13 rare-earth complexes, [NEt_4_]_3_[LnCl_2_(MeCN)­{(μ-S)_2_WS_2_}_2_] (**1Ln**, Ln = Ce–Yb and Y). The unusually low coordination numbers (CN = 7) observed here in the absence of sterically bulky or rigid chelating ligands was rationalized using buried volume analysis. We highlight the potential of this new ligand set for luminescent and single-molecule magnets applications by investigating the properties of **1Yb** and **1Dy** complexes, respectively.

## Graphical abstract

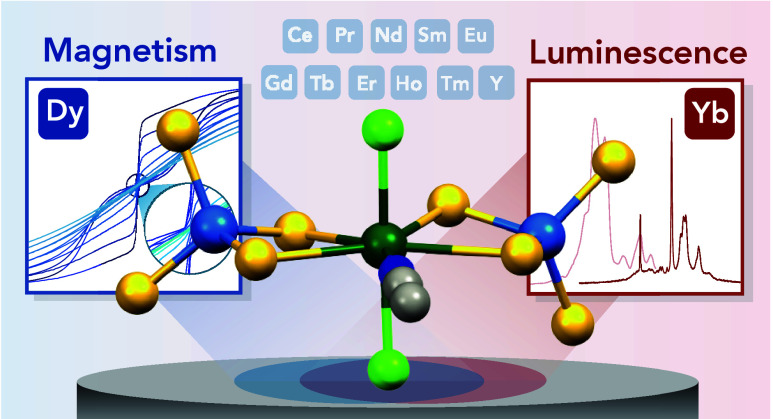

## Introduction

Lanthanides exhibit a unique coordination chemistry due to the shielding of their valence electrons in the 4f orbitals, which imparts a highly ionic character, where interactions between Ln^3+^ cations and ligands are largely electrostatic. The coordination numbers (CNs) and complex geometries are therefore determined by steric factors, resulting in high CNs, with examples of CN < 8 being the exception rather than the norm in the literature.

These hard ions preferentially bind to hard donors, such as oxygen, nitrogen and carbon, which constitute approximately 95% of the donor atoms in lanthanide complexes according to data from the Cambridge Structural Database.^1^ Due to the electrostatic and nondirectional nature of the Ln–ligand interactions, achieving lower coordination numbers typically requires steric control in the second coordination sphere, often through the use of large organic ligands.^2,3^ However, this approach can impede luminescence and magnetic properties due to an increased amount of X–H (X = O, N, C) vibrational oscillators and stability issues,^4^ highlighting the need for innovative strategies to achieve lower coordination numbers without relying on complex organic architectures. In this context, we aimed to explore the possibility of achieving low coordination numbers by shifting the steric control to the primary coordination sphere of lanthanide ions using larger donor atoms, specifically sulfur-based ligands, thus bypassing the need for bulky organic substituents to increase the overall steric bulk (Figure 1). However, due to the oxophilic nature of lanthanides, transitioning from oxygen- to sulfur-based donors presents inherent synthetic challenges, complicating the transmetalation from common lanthanide precursors to softer Lewis bases, such as thiolate or sulfido ligands.^5,6^

**Figure 1:**
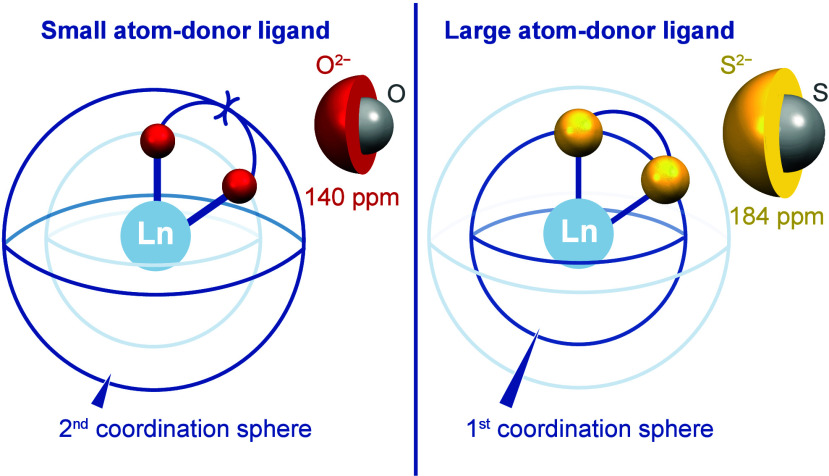
Visual representation of how the local geometry for rare-earth complexes can be controlled, using steric bulk in either the second coordination sphere in the case of small-atom-donor ligands or in the first coordination sphere when using large-atom-donor ligands.

Despite the softness of sulfur-based ligands, a few remarkable lanthanide-based complexes have been reported with unique luminescent and magnetic properties.^7–16^ While these strategies successfully demonstrated the possibility of modulating Ln properties with weak-field sulfide ions, they still relied on organic-based ligands to control the coordination sphere of the Ln ion. In this context, we sought to explore the use of fully inorganic sulfur-based ligands to prepare lanthanide complexes. We recently demonstrated that tetrathiotungstates can serve as the sole ligands for trivalent lanthanide ions, enabling the separation of Eu from complex lanthanide mixtures in spent fluorescent lamps with unprecedented separation factors.^17^ Since the isolation of the first discrete thiometalato complex, [Ni­(WS_4_)_2_]^2–^, in 1971, tetrathiometallate anions [MS_4_]^2–^ (M = Mo, W) have been proven as versatile building blocks to synthesize inorganic heterometallic clusters when combined with transition metals.^18–22^ Yet, these anions have been essentially unexplored for Ln ions, despite being reported as promising bridging ligands to promote magnetic exchange in the bimetallic single-molecule magnet complexes [PPh_4_]­[(Cp*_2_Sm)_2_Mo­(μ-S)_4_] and [PPh_4_]­[Cp*_2_Sm­(μ-S)_2_WS_2_].^23,24^

In the present work, we demonstrate that tetrathiotungstate anions enable the preparation of fully inorganic, low-coordination-number lanthanide chalcogenide complexes. This unique inorganic coordination environment unlocks new strategies to design magnetic and luminescent molecules. We synthesized, structurally characterized, and spectroscopically analyzed a series of 13 isostructural lanthanide complexes bearing two tetrathiotungstate ligands, with the general formula [NEt_4_]_3_[LnCl_2_(MeCN)­{(μ-S)_2_WS_2_}_2_] (**1Ln**, Ln = Ce–Yb and Y). Due to its larger size, La afforded the dimer [NEt_4_]_6_[La­(μ_2_-Cl)_2_{(μ_2_-S­(μ-S)_2_WS}­{(μ-S)_2_WS_2_}_2_]_2_ (**2La**).

In addition to their singular structural and bonding properties, we selected two members of the series to illustrate the capacities of this new ligand set combined with the lanthanides of interest in single-molecule magnet and optical applications: **1Dy** displayed promising SMM properties, displaying an open magnetic hysteresis up to 7 K and an anisotropy barrier of 260 cm^–1^, while **1Yb** demonstrated remarkable near-infrared-emitting properties of the Yb analogue with an exceptionally sharp spectral profile, reminiscent of luminescence stemming from inorganic materials. These selected examples illustrate the potential of the WS_4_^2–^ ligand for magnetic and luminescence application: the fully inorganic character of the ligands minimizes the vibrational contribution to the nonradiative deactivation processes, and the unique pairing of soft donor sulfur atoms in the equatorial plane with hard chloride donors along the axial direction, dramatically enhances the axial magnetic anisotropy and effectively control the local symmetry to limit zero-field quantum tunneling.^25–28^

## Results and Discussion

### Synthesis and Structural Characterization

Our first successful attempt to synthesize heteropolymetallic assemblies of rare earths and tetrathiometallates resulted from the reaction between EuCl_3_ and (NEt_4_)_2_WS_4_ in MeCN (1:2 ratio). Upon addition of europium chloride to the tetrathiotungstate solution, an immediate color change from bright yellow to dark red is observed. After 2 h stirring at room temperature, vapor diffusion of Et_2_O at −35 °C leads to the formation of two types of crystals with different morphologies: colorless hexagonal prisms coexist with orange plates. Single-crystal XRD analysis of the latter reveals the formation of the trinuclear species [NEt_4_]_3_[EuCl_2_(MeCN)­{(μ-S)_2_WS_2_}_2_] (**1Eu**), while the colorless crystals were determined to be (NEt_4_)_3_Eu_2_Cl_9_ (Figure 2a). It is notable that EuCl_3_ acts as a chloride abstractor in this reaction to afford the dimeric complex [Eu_2_Cl_9_]^3–^.^29^

**Figure 2:**
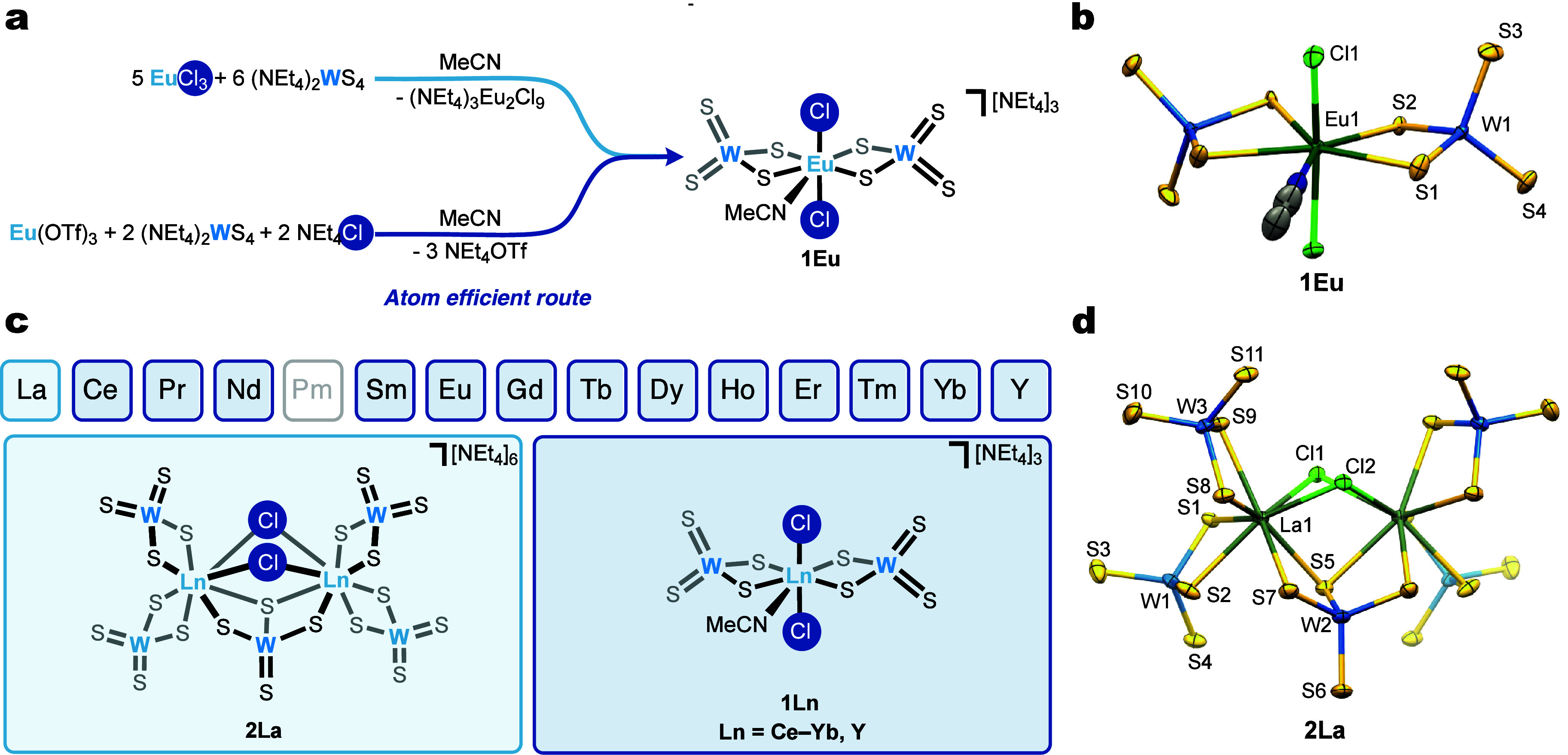
(a) Synthesis of **1Eu** via two possible routes. (b) Crystal structure of **1Eu** with thermal ellipsoids shown at the 50% probability level. (c) Structures of the lanthanum dimer **2La** and of the series **1Ln** (Ln = Ce–Yb and Y). (d) Crystal structure of **2La** with thermal ellipsoids shown at the 50% probability level. Hydrogen atoms and counter-cations are omitted for the sake of clarity.

From isolated solutions of **1Eu**, we were also able to observe the formation of the previously reported reduced coordination polymer ([NEt_4_]_2_[Eu^II^(WS_4_)_2_])_n_ at longer reaction times, demonstrating that the presence of halides slows down but does not prevent the internal electron transfer induced reduction of Eu.^17^ Abstraction of the chloride ions from **1Eu** in the presence of strong Cl^–^ scavengers such as (OEt_3_)­BF_4_ or NaBArF proved unsuccessful, affording solely decomposition products and the formation of the trinuclear tungsten cluster (NEt_4_)_2_W_3_S_9_.^30,31^ To circumvent the use of excess lanthanide as halide abstractor and provide a more straightforward synthetic route, we adapted the stoichiometry of the reaction to the targeted complex via the reaction of Eu­(OTf)_3_ in the presence of 2 equiv of (NEt_4_)_2_WS_4_ and NEt_4_Cl, the latter acting both as halide donor and countercation.

This simplified synthetic route affords the desired complex **1Eu** in 66% yield (Figure 2a,b) and can be directly applied to other rare earths, and the complexes [NEt_4_]_3_[LnCl_2_(MeCN)­{(μ-S)_2_WS_2_}_2_] (**1Ln**, Ln = Ce–Yb and Y) can be isolated as light yellow to dark red crystalline solids in higher than 60% yields (Figure 2c). This constitutes the first isostructural series of 13 rare-earth complexes bearing soft donor ligands. The lanthanide ions are coordinated by two k^2^-tetrathiotungstate ligands in the equatorial plane, two axial chloride ligands and one acetonitrile solvent molecule to reach a rather unusual low coordination number CN = 7. Size contraction of the lanthanide ions can be observed across the series, resulting in distortion in the equatorial plane to accommodate the smaller lanthanides, as illustrated by the variation of the S1–Ln–S1′ angles across the series (from 145.29(7)° for **1Ce** to 140.27(5)° for **1Yb**). Consequently, larger ions crystallize in the monoclinic *C*2/*c* space group, with a mirror plane along the MeCN ligand (**1Ce** to **1Dy**), while smaller ions crystallize in a lower symmetry, namely triclinic *P*1 (**1Ho** to **1Yb** and **1Y**). The absence of lanthanum from this series is notable, and under the same reaction conditions the preferred structure for the largest 4f ion is the dimeric structure [NEt_4_]_6_[La­(μ^2^-Cl)_2_{(μ^2^-S­(μ-S)_2_WS}­{(μ-S)_2_WS_2_}_2_]_2_ (**2La**) (Figure 2d). Worthy of note, examination of molecular packing revealed that a rich array of intermolecular hydrogen bonds is involved in the crystal packing,^32^ with eight C–H···S from the terminal sulfides and two C–H···Cl interactions per molecule for **1Ce** (with C–H···S angles above 155° and C···S distance below 5 Å). This interaction exhibits one of the shortest C–H···S contacts ever reported between the CH_3_ group of the bound acetonitrile and a terminal sulfide (d­(C–S) = 3.722 Å, and a C–H···S angle of 162.39°),^33^ highlighting the overall high negative charge of the complex. For smaller lanthanides, the packing involved fewer hydrogen bonds with four C–H···S and two C–H···Cl interactions per molecule for **1Yb** (d­(C–S) = 3.843 Å, and a C–H···S angle of 163.85°), in good agreement with their higher Lewis acidity, overall lowering the electron density at the terminal sulfides (Figure 3a)

**Figure 3:**
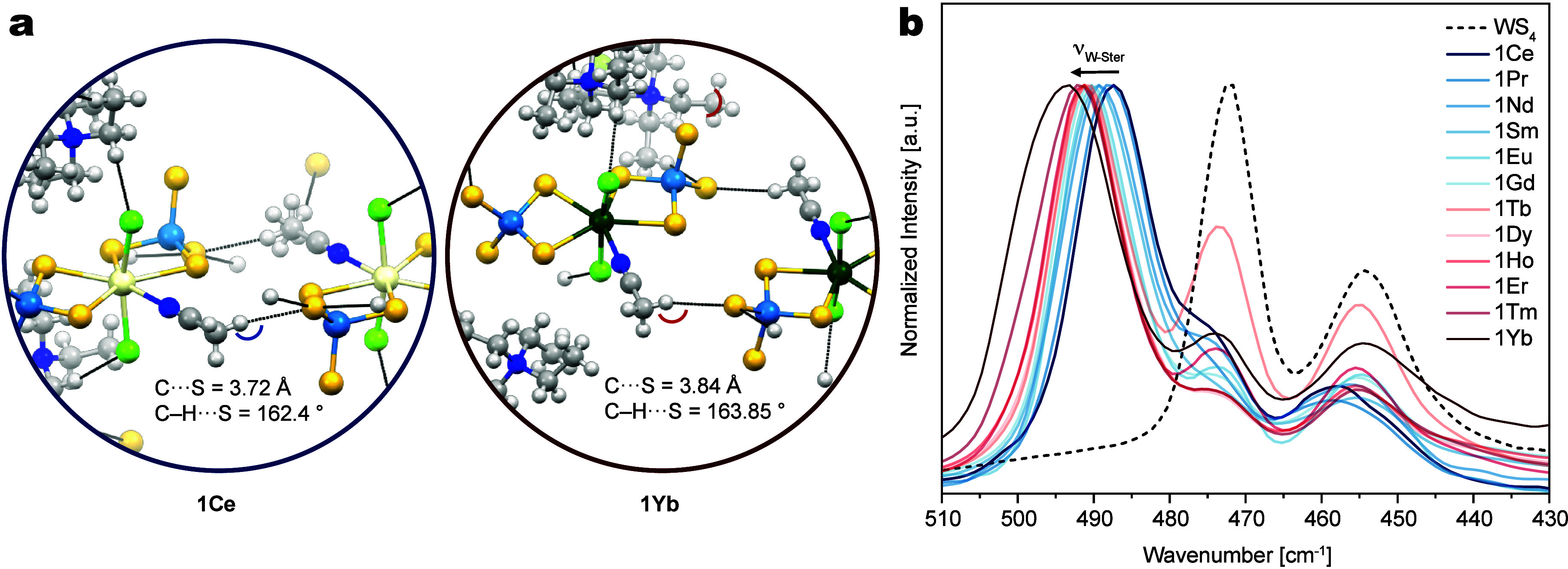
(a) Intermolecular crystal packing and H-bond network in **1Ce** and **1Yb**. (b) Normalized Raman spectra in MeCN of **1Ln** (Ln = Ce–Yb) compared to the free WS_4_^2–^ ligand.

Independently of the space group, the local Ln ion geometry of all monomeric Ln complexes is best described as a pentagonal bipyramid (*D*_5*h*_) according to a SHAPE analysis of the Ln coordination sphere (SHAPE 2.1), revealing continuous shape measure (CShM) values for this geometry range between a minimal of 1.098 for **1Dy** and a maximum of 1.150 for **1Yb**.^34–37^ The Ln–S bonds are usual and comparable to those reported for dithiocarbamate complexes,^38–40^ following an expected shortening along Ln ion contraction across the series, from Ln–S1 = 3.0109(18) Å and Ln–S2 = 2.9391(16) Å for **1Ce** down to Ln– S1 = 2.9196(19) Å and Ln–S2 = 2.8022(17) Å for **1Yb**. However, coordination of the tetrathiotungstate to the Ln ion induces significant structural modifications within the ligand moiety itself. Compared to the relatively uniform W–S bond lengths in the free tetrathiotungstate, which range from 2.1846(12) to 2.2030(12) Å,^41^ the **1Ln** complexes exhibit a clear desymmetrization of the ligand. Indeed, the bridging sulfides (S_bri_) display elongated bond lengths, while the terminal sulfides (S_ter_) show noticeably shortened bond lengths. More specifically, across the series, the W–S_bri_ distances span from 2.2203(15) and 2.2000(18) Å for **1Ce** to 2.2197(17) and 2.1968(18) Å for **1Yb**, while the W–S_ter_ distances range from 2.2175(2) and 2.1712(17) Å for **1Ce** and 2.181(2) and 2.1700(18) Å for **1Yb**.

These structural features are also reflected in the Raman spectra of the complexes: while the free ligand exhibits characteristic W–S vibrations at 472 cm^–1^ (ν1­(A1)), 454 cm^–1^ (ν3­(F2)), and 174 cm^–1^ (ν2­(E)),^42^ a new vibrational band emerges near 490 cm^–1^ upon coordination to the lanthanide center which is red-shifted from 487.4 cm^–1^ for **1Ce** to 493 cm^–1^ for **1Yb** (Figure 3b). This trend correlates with the increasing Lewis acidity of the Ln ion, which in turn strengthens the W–Ster bond upon coordination. The Ln–Cl bonds span from 2.713(1)) Å for **1Ce** to 2.541(7) Å for **1Yb** across the series, in good agreement with the progressive shortening expected from the decreasing ionic radii of the Ln centers along the series. The red shift in the Raman spectra of the vibrational band at 238 cm^–1^, observed from **1Tb** to **1Yb** and assigned to the Ln–Cl vibration,^43^ further confirms this trend (Supporting Information Figure S17).

### Buried Volume Analysis

In contrast to transition-metal complexes, the coordination number in lanthanide complexes is primarily governed by the steric crowding within the coordination sphere, owing to the predominantly electrostatic nature of the Ln–ligand interactions.^44^ Buried volume analysis, as developed by Cavallo and co-workers,^45–47^ therefore appears as a powerful tool to evaluate the steric control of the coordination sphere in lanthanide complexes, and was proven effective to correlate dimerization trends.^48^ Here, we further exploited buried volume analysis to better discriminate between the steric contributions of the primary and secondary coordination spheres in the **1Ln** series. The buried volume (%V_bur_) was calculated using the SambVca 2 web tool with the.*xyz* files for all **1Ln** single-crystal XRD structures.^46,47^ The Ln center was chosen as the center of the sphere. The nitrogen atom of the bound acetonitrile ligand (N1) was chosen as the *z* axis definition (Z-positive). The bridging sulfur facing the acetonitrile ligand (S1) was chosen as the atom for the *xz*-plane definition. The metal center was then deleted. The bond radii were not scaled and the sphere radius was set to different *R* values. By systematically varying the buried volume sphere size from 2.5 to 5 Å, we identified an inflection point in %V_bur_ at *R* = 3 Å, which allowed us to define %V_bur1_ as the buried volume corresponding to the first coordination sphere, while the convergence of the buried volume at *R* = 5 Å enabled to define %V_bur2_ for the second coordination sphere (Figure 4a).

**Figure 4:**
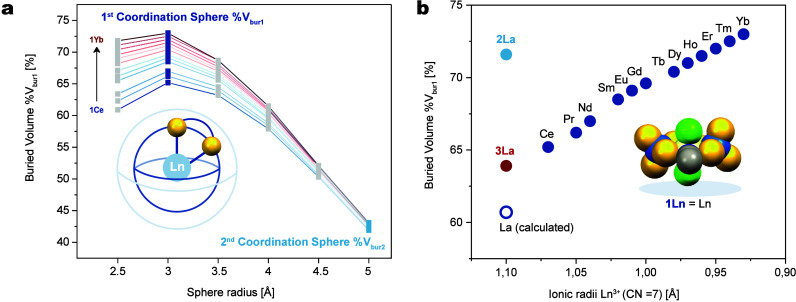
(a) Plot of the %V_bur_ for **1Ln** (Ln = Ce–Yb) with varying size of the sphere from *R* = 2.5 Å to *R* = 5 Å. (b) Plot of the %V_bur1_ as a function of the ionic radii for **1Ln** (Ln = Ce–Yb; dark blue circles), **2La** (light blue circle), and **3La** (red circle) and DFT-optimized structure of the hypothetical monomeric complex **1La** (dark blue empty circle).

Looking at the first coordination sphere as defined above, %V_bur1_ has values ranging from 65.2 for **1Ce** to 73 for **1Yb**, an increase consistent with the linear decrease of the ionic radii across the series, whereas the complex **2La** shows that La can accommodate more steric bulk in its first coordination sphere with %V_bur1_ = 71.6. This illustrates a threshold in the buried volume below which the coordinatively unsaturated metal center dimerizes to increase its coordination number from CN = 7 to CN = 8.

Reasoning on the fact that buried volume and associated accessible coordination space is the drive for the dimerization of the lanthanum complex, we attempted replacing the chloride anions by larger bromides. The isostructural complex with axial bromide ligands, [NEt_4_]_3_[LnBr_2_(MeCN)­{(μ-S)_2_WS_2_}_2_] (**3La**), could accordingly be synthesized, using NEt_4_Br instead of NEt_4_Cl in an exactly analogous synthetic procedure. To the best of our knowledge, **3La** is a rare example of a seven-coordinate lanthanum complex which does not require sterically bulky ligands.^44^ A comparison of the optimized geometry parameters of **3La** and the hypothetical **1La** showed smaller values of %V_bur1_ for the latter.

This indicates that the threshold for dimerization resides on a narrow geometry window—a notion further supported by the fact that the %V_bur1_ of **3La** aligns fairly well with a linear correlation of the values for **1Ln** (Ln = Ce–Yb). Interestingly, the discrimination between the first and the second coordination sphere buried volumes enables a clear distinction between the approach developed here, using tetrathiotungstate ligands, and previously reported complexes in the literature with the same coordination number (CN = 7). Traditional bidentate O–N donor ligands, commonly used to stabilize seven-coordinated complexes, tend to exhibit a relatively small difference in buried volume between the first and second coordination spheres. This can be explained by the need to have a sterically crowded second coordination sphere to enforce lower coordination numbers. In contrast, the use of tetrathiotungstate ligands allows control of the coordination number by playing only on the first coordination sphere, as illustrated by the large difference between %V_bur1_ and %V_bur2_. This can be further visualized using the steric map tool available on the Sambvca Web site, which reveals that the second coordination sphere of **1Dy** is significantly less encumbered compared to other seven-coordinated bis-chloride complexes (examples 1–3) and only large planar chelating ligands can reach similar geometrical constrains (examples 4–6) (Table S8).^45,46,49^

### Luminescence Properties

Following crystal field theory, highly asymmetric lanthanide complexes can cause exceptions to the forbidden selection rules in 4f–4f transitions, promoting intense luminescence of the complexes with increased observed lifetimes. In that regard, seven-coordinated complexes have been considered interesting targets to design luminescent molecules.^50^ Accordingly, we explored here the luminescence properties of the ytterbium complex **1Yb**. The photoexcitation of the crystalline **1Yb** complex at λ_exc_ = 360 nm demonstrated characteristic near-infrared emission corresponding to the ^2^F_5/2_→^2^F_7/2_ transition of Yb^3+^ ions (Figure 5a, Figure S28). At room temperature, the emission spectral profile comprised several hot bands below 975 nm, originating from thermally populated excited states,^51^ which almost completely disappeared upon cooling to 77 K (Figure 5a, blue). At low temperature, the emission spectrum represented exceptionally well-resolved and sharp signals usually observed for inorganic materials doped with Yb^3+^ ions.^52^ The corresponding excitation spectrum was recorded at λ_em_ = 1050 nm at 77 K to determine the zero-phonon line of the ^2^F_5/2_→^2^F_7/2_ transition,^53^ located at 985 nm (10153 cm^–1^) (Figure 5a, black), and the crystal field splitting of the Yb^3+^ excited state, making up to 481 cm^–1^. The four emission bands of the **1Yb** crystals at 77 K permitted the experimental estimation of the ground state crystal field splitting, the total energy of which was equal to 560 cm^–1^ (Figure 5b). As commonly observed for Yb^3+^ complexes, the calculated CASPT2 energies of the Kramers doublets of the ground state (^2^F_7/2_) and first excited state (^2^F_5/2_) multiplets align well with the experimental results from the luminescence spectra (Figure 5b and Table S12).^54^

**Figure 5:**
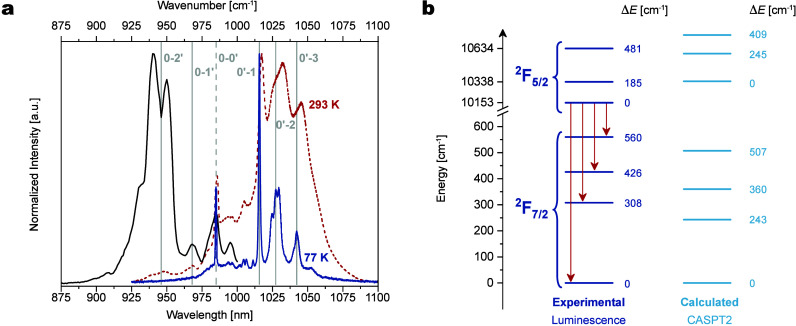
(a) Steady-state excitation (λ_em_ = 1050 nm; black, 77 K) and emission spectra (λ_exc_ = 360 nm; red dashed, 293 K; blue, 77 K) of **1Yb** crystals. The vertical gray lines correspond to the experimental energy splitting of the ground and excited states; the dashed line is a zero-phonon line. (b) Energy diagram summarizing experimental and calculated energy splitting of the ground ^2^F_7/2_ and excited ^2^F_5/2_ states of Yb^3+^ ions in **1Yb**.

The emission decay of **1Yb** modeled into a monoexponential fit yielding 10.7 μs observed lifetime in solid state at 293 K (Figure S29). This study clearly shows that tetrathiotungstate ligands can act as purely inorganic antennae deprived of any organic conjugated system for the sensitization of Yb^3+^ luminescence. Furthermore, the almost complete absence of organic X–H oscillators in the vicinity of the emitting center results in very narrow emission signals at low temperature for a molecular complex, similar to those generally observed for bulk inorganic materials. In conclusion, the tetrathiotungstate ligand is a promising antenna that can sensitize the near-infrared Yb^3+^ emission, permitting to explore the lanthanide luminescence properties in this particular coordination environment.

### Magnetic Properties

Magnetic anisotropy has played a key role to improve the physical properties of single-molecule magnets. In 2011, Rinehart and Long proposed a simple electrostatic model to explain the magnetic properties of the Ln^3+^ ions, based on the angular dependence of their electronic density anisotropy. This model allows to strategically maximize the magnetic anisotropy by choosing the ligands and the geometry of the lanthanide coordination site.^55^ In the case of oblate ions such as Dy^3+^, low symmetry geometries with strong axiality should maximize the magnetic anisotropy. This favorable geometry, further enhanced by the combination of strong axial chloride ligands and softer sulfur donors in the equatorial plane, prompted us to investigate the magnetic properties of **1Dy**. At room temperature, χ_M_ *T*, with χ_M_ the molar magnetic susceptibility and *T* the temperature in kelvin, is equal to 13.9 cm^3^ K mol^–1^ close to the expected value for an isolated ^6^H_15/2_ multiplet (Figure S30).

On cooling, χ*M* *T* decreases smoothly due to the thermal depopulation of the crystal field states down to 12 cm^3^ K mol^–1^ at 10 K, then drops more rapidly to 11 cm^3^ K mol^–1^ at 2 K (Figure S30). This behavior is attributed to the combined influences of out-of-equilibrium magnetization and possible intermolecular interactions.

CASSCF calculations confirm this trend, accurately reproducing the experimental χ_M_ *T* vs *T* curve above 10 K. The magnetization curve measured at 10 K is also well reproduced by the calculations (Figure S30). The ground Kramers doublet state is a pure *M*_J_ = |±15/2⟩ with *g* values in the effective spin 1/2 Hamiltonian framework equal to 0.0, 0.0, and 19.9 for *g*_*x*_, *g*_*y*_, and *g*_*z*_, respectively (Table S14). The calculated easy axis (*z* axis) is, as expected for an oblate electronic distribution, oriented toward the harder donor atoms (Figure 5b). The first excited Kramers doublet state (pure |±13/2⟩) is localized at 203 cm^–1^ above the ground state. AC susceptibility measurements in zero external DC field confirm the Ising nature of the Kramers doublet ground state. The frequency dependence of the out-of-phase component of the AC susceptibility is clearly visible below 24 K (Figure 6a). The relaxation time was extracted at each temperature from extended Debye data treatment (Table S10 and Figures S31 and S32) and is represented as an Arrhenius plot on Figure 5b. The thermal variation of the relaxation time is analyzed with the combination of three different processes, namely Orbach, Raman, and quantum tunneling of magnetization, each dominant in three distinct temperature ranges (Figure 6b). The extracted barrier of 595 cm^–1^ is in good agreement with the calculated transition moments that support the thermally activated Orbach relaxation process via second or higher excited states (Figure 6b, Figure S35). At very low temperatures, quantum tunneling of magnetization becomes the dominant process. The magnetic properties of the Dy complex can hence be improved upon dilution in an isomorphous diamagnetic matrix of the Y analogue (5:95), **1Dy@Y**: at zero field the relaxation time in the quantum regime is about ten times slower for **1Dy@Y** compared to **1Dy** (Figure 6a, Figure S33). Data treatment using the extended Debye model for **1Dy@Y** (Table S11) reveals that the Raman and Orbach contributions to the relaxation process remain very similar to **1Dy** (the energy barrier remains the same at 260 cm^–1^), while only the quantum regime is affected, slowed down, by the dilution (Figure 6b). As a result, the hysteresis loop of the diluted sample exhibits remanence in zero external field up to 7 K, whereas it remains closed for **1Dy** (Figure 6c and Figure S34).

**Figure 6:**
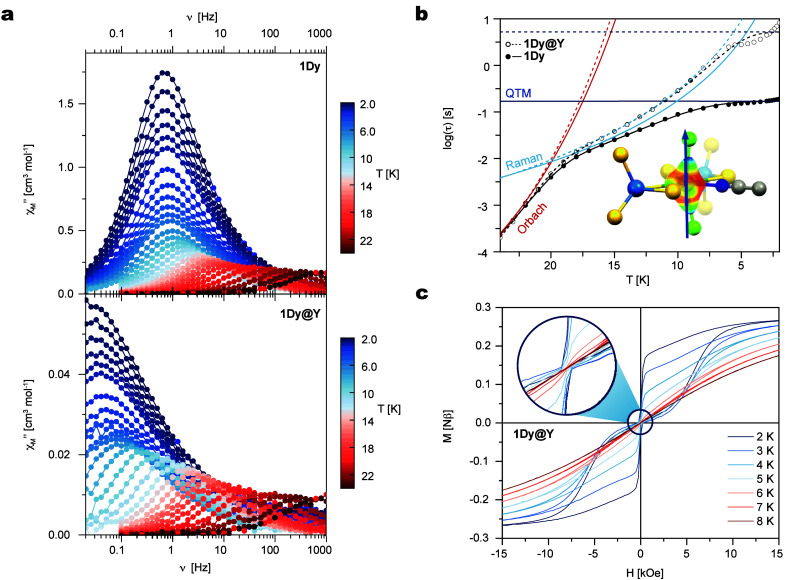
(a) Frequency dependence of the out-of-phase component of the AC (alternating current) susceptibility measured in zero external DC (direct current) field in the condensed phase **1Dy** (top) and in the dilute 95:5 yttrium matrix **1Dy@Y** (bottom). (b) Thermal dependence of log­(*t*) for **1Dy** (black dots) and **1Dy@Y** (white dots) under an applied magnetic field (full lines for **1Dy** and dotted lines for **1Dy@Y**). Orbach, Raman, and quantum tunneling of magnetization (QTM) contributions are shown in orange, green, and blue, respectively. Inset: orientation of the calculated magnetic easy axis of the Kramers doublet ground state for **1Dy** onto the calculated total electrostatic potential (expressed in e^–^ bohr^–1^) at 2 Å. (c) Hysteresis curves of the dilute matrix of **1Dy@Y**. Inset: zoom in on the zero-field region, illustrating the opening on the hysteresis in zero external field.

The promising magnetic properties of the complex further illustrate a distinctive feature of the tetrathiotunsgtate ligands. These enable a significant difference in the electron-donating character between the axial and equatorial plane of the Dy center, combining hard halides in the axial position with soft sulfur donors in the equatorial plane. This arrangement leverages the advantages of the pentagonal bipyramid geometry by ensuring the principal axis is aligned with the main magnetic axis, thereby maximizing the magnetic anisotropy, a condition that is more critical than the geometry itself.^56^ This approach strongly contrasts with the reported literature examples of Dy^3+^ complexes featuring pentagonal bipyramid geometries with axial chloride ligands. As summarized in Table S9, previous strategies relied on either bulky bidentate^57–59^ or large planar polydentate organic ligands bearing hard donor atoms (O, N). These designs generally failed to sufficiently differentiate the electronic properties of the axial Cl ligands from those of the equatorial donors, preventing the establishment of a strong axial magnetic axis.^60–64^

## Conclusion

In the present work, we illustrate that the use of tetrathiometallates enables the preparation of a complete series of fully inorganic lanthanide chalcogenide complexes. The use of large sulfur donors allows to precisely control the coordination geometry of the lanthanide ion, circumventing the need for bulky organic substituents in the second coordination sphere to impose low coordination numbers. Most importantly, the use of tetrathiotungstate ligands demonstrated remarkable luminescent and magnetic performances of lanthanide ions, which are untrivial to achieve in molecular systems. In particular, the all-inorganic nature of the ligands prevented quenching by vibrational oscillators, increasing the observed luminescence lifetime, and the combination of soft sulfur donors in the equatorial plane and hard halide donors in the axial plane were found key to improving anisotropy and afford high blocking temperatures in oblate ions such as dysprosium. Further investigations are currently underway to extend this ligand platform across this series and beyond, in order to further explore the potential of this family of inorganic sulfur donors.

## Experimental Section

### General Considerations

*Elemental analyses* were carried out at the Molecular and Biomolecular Analysis Service (MoBiAS) of ETH Zürich on a LECO TruSpec Micro spectrometer. *NMR* data were recorded on a 200 MHz Bruker Avance II spectrometer at room temperature. ^1^H spectra are reported in parts per million (ppm) and are calibrated with respect to the corresponding solvent residual peak. *Raman spectroscopy* data were collected using a Thermo Scientific DXR Smart Raman spectrometer and processed with the OMNIC software. Spectra were obtained using a 532 nm excitation laser wavelength, 10 mW output power and a spectral resolution of 1 cm^–1^. Samples were measured in NMR tubes sealed with a J. Young valve. *Single crystal X-ray* data were collected on a Rigaku XtaLAB Synergy-S diffractometer equipped with a HyPix-6000HE detector using Cu Kα radiation (λ = 1.54184 Å) at 100 K. After data collection, structures were solved by intrinsic phasing (SHELXT) and refined by full-matrix least-squares procedures on F2 using SHELXL in the olex2 program suite.^65–67^ All non-hydrogen atoms were refined with anisotropic displacement parameters. The hydrogen atoms were placed in positions of optimized geometry. *Continuous shape measures* (CShM) were calculated using the SHAPE 2.1 Software with the.xyz files for all **1Ln** structures.^35–37^ *Buried volume* (%V_bur_) was calculated using the SambVca 2 web tool with the .xyz files for all **1Ln** structures.^46,47^ The Ln center was chosen as the center of the sphere. The nitrogen atom of the bound acetonitrile ligand (N1) was chosen as the *z* axis definition (Z-positive). The bridging sulfur facing the acetonitrile ligand (S1) was chosen as the atom for the *xz*-plane definition. The metal center was then deleted. The bond radii were not scaled, and the sphere radius was set to different *R* values. The distance of the coordination point from the center of the sphere was set to 0.0 and the mesh spacing for numerical integration was set to 0.10.

*The luminescence properties* were measured using Horiba–Jobin–Yvon Fluorolog-3 fluorometers. The crystalline **1Yb** samples in sealed J. Young tubes were excited by unpolarized light from a 450 W xenon continuous wave lamp and detected at right angle through a FGL850 high-pass filter by using a liquid nitrogen cooled Symphony II CCD-camera iHR320 series (emission spectra) or a solid indium/gallium/arsenic detector (850–1600 nm, excitation spectra). Spectra were corrected for both excitation source light–intensity variation (lamp and grating) and emission spectral responses (detector and grating). The spectra at 77 K were measured by submerging the sealed tubes in a liquid-nitrogen-filled quartz Dewar flask. *The luminescence lifetimes* of **1Yb** complex were measured using a pulsed Nd:YAG laser (SpectraPhysics), operating at 10 Hz. Light emitted at right angles to the excitation beam was focused onto the slits of a monochromator (PTI120), which was used to select the appropriate wavelength. The growth and decay of the luminescence at selected wavelengths was detected using a Ge photodiode (Edinburgh Instruments, EI-P) and recorded using a digital oscilloscope (Tektronix TDS320) before being transferred for analysis. Luminescence lifetimes were obtained by iterative reconvolution of the detector response (obtained by using a scatterer) with exponential components for growth and decay of the metal-centered luminescence. The DC and AC magnetic susceptibility measurements were performed on solid polycrystalline samples with a Quantum Design MPMS-XL SQUID magnetometer between 2 and 300 K in an applied magnetic field of 0.02 T in the temperature range 2–20 K, 0.2 T in the temperature range 18–60 K, and 1 T for temperatures above 60 K. All samples have been prepared in a glovebox and measured in sealed EPR tubes to preserve their integrity. To ensure that the powder does not orientate with dc field, we prepared small pellets of powder with PTFE tape. The experimental data have been corrected from the diamagnetism of the sample holder (PTFE+EPR tube), and the intrinsic diamagnetism of the materials was evaluated with Pascal’s tables. The hysteresis loops at ^3^He temperatures have been measured with a ^3^He insert (iHelium3) adapted to a SQUID magnetometer. The magnetic field is then swept in hysteresis mode and the magnetic moment measured with RSO head. The measurement time at each field is then close to 20 s.

### Synthesis

#### Materials

Unless stated otherwise, syntheses were carried out under strict inert argon atmosphere using Schlenk techniques or inside Vigor gloveboxes. Pentane was stirred over concentrated sulfuric acid, rinsed with aqueous bicarbonate solution and deionized water, and dried over calcium chloride beads before being used in a solvent purification system. Diethyl ether pentane and acetonitrile were dried using a Vigor solvent purification system. Diethyl ether was additionally dried over potassium/benzophenone, distilled, degassed by three freeze–pump–thaw cycles, and stored over 4 Å molecular sieves for at least 3 days prior use. Likewise, pentane and acetonitrile were degassed by three freeze–pump–thaw cycles and stored over 4 and 3 Å molecular sieves, respectively, prior use. H_2_WO_4_ was purchased from Fluka. Lanthanum­(III) oxide (La_2_O_3_, 99.99% trace metals basis) was purchased from ABCR. Cerium­(IV) oxide (CeO_2_, 99.99% trace metals basis), praseodymium­(III,IV) oxide and samarium­(III) oxide (Sm_2_O_3_, > 99.8% trace metals basis) were purchased from Fluka. Neodymium­(III) oxide (Nd_2_O_3_, 99.99% trace metals basis), terbium­(III) oxide (Tb_2_O_3_, 99.99% trace metals basis), and erbium­(III) oxide (Er_2_O_3_, 99.99% trace metals basis) were purchased from Sigma-Aldrich. Gadolinium­(III) oxide (Gd_2_O_3_, 99.99% trace metals basis), dysprosium­(III) oxide (Dy_2_O_3_, 99.9% trace metals basis), holmium­(III) oxide (Ho_2_O_3_, 99.9% trace metals basis), and thulium­(III) oxide (Tm_2_O_3_, 99.99% trace metals basis) were purchased from Alfa Aesar. Europium­(III) oxide (Eu_2_O_3_, 99.99% trace metals basis) and ytterbium­(III) oxide (Yb_2_O_3_, 99%) were purchased from Acros Organics. Yttrium­(III) oxide (Y_2_O_3_, 99.99%) was purchased from Ventron. Trifluoromethanesulfonic acid (99%) was purchased from Apollo Scientific Ltd. Tetraethylammonium hydroxide (25% in water) was purchased from Acros Organics. Tetraethylammonium chloride (>98%) was purchased from Sigma-Aldrich, recrystallized from EtOH/Et_2_O, and dried under high vacuum. Triethyloxonium tetrafluoroborate (OEt_3_)­BF_4_ (>97%) was purchased from Fluka, and NaBArF was synthesized according to literature procedure.^68^ (NH_4_)_2_WS_4_ and (NEt_4_)_2_WS_4_ were synthesized according to modified literature procedure,^69^ using either an H_2_S gas bottle (99.5%) from Air Liquide or generating H_2_S in situ using a Kipps apparatus filled with FeS fused sticks from Merck Millipore and sulfuric acid (H_2_SO_4_, 95–98%) from Sigma-Aldrich.

#### Synthesis of (NH_4_)_2_WS_4_

(NH_4_)_2_WS_4_ was synthesized according to the following modified literature procedure.^69^

The synthesis was performed in a well ventilated fumehood.

H_2_WO_4_ (10 g, 40 mmol) was dissolved in aqueous NH_3_ (25%, 60 mL), and the solution was constantly purged with H_2_S, bubbling through the solution via a Teflon canula. While maintaining the constant H_2_S purge and stirring the solution, color changes from pale white over lime-green to bright green were observed, and after 1 h, the solution was heated gradually to 60 °C over a period of 3 h, cooled to room temperature, and stirred overnight. After that time the formation of a yellow solid was observed. The solid product was isolated by filtration, washed with ^i^PrOH (3 × 25 mL) and Et_2_O (3 × 25 mL), and dried in vacuo to give a yellow powder (10.91 g, 31.3 mmol, 78%). Elem. Anal. Found (calcd), %, for H_8_N_2_WS_4_: H, 2.38 (2.32); N, 8.24 (8.05). UV–vis (H_2_O, 0.9 × 10^–4^ M; 1 cm path); λ_max_(nm) (ε (M^–1^ cm^–1^)): 216 (32740), 278 (28448), 393 (19247).

Note: Excess H_2_S gas was quenched using gas-wash bottles in series, filled first with sodium hypochlorite followed by 1 M KOH.

#### Synthesis of (NEt_4_)_2_WS_4_

(NEt_4_)_2_WS_4_ was synthesized according to the following modified literature procedure.^69^

(NH_4_)_2_WS_4_ (4.9 g, 14 mmol) was dissolved in NEt_4_OH (25% in H_2_O, 16 mL) and degassed H_2_O (20 mL). The solution was subjected to pumping for 2 h while stirring, before ^i^PrOH (150 mL) was added, resulting in the formation of a bright yellow precipitate, which was allowed to settle at 0 °C. The supernatant was removed by cannula filtration and the solids were washed with ^i^PrOH (2 × 50 mL) and Et_2_O (2 × 50 mL) and dried in vacuo overnight. The crude product was extracted with MeCN and dried in vacuo overnight, affording the title complex as bright yellow crystals (6.06 g, 10.6 mmol, 75%). Elem. Anal. Found (calcd), %, for C_16_H_40_N_2_WS_4_: C, 33.60 (33.56); H, 7.03 (7.04); N, 5.10 (4.89). UV–vis (MeCN, 1.9·10^–4^ M, 2 mm path) λ_max_(nm) (*ε* (M^–1^ cm^–1^)): 223 (28 844); 284 (25 290); 399 (19 534).

#### Synthesis of Ln­(OTf)_3_ (Ln = La–Yb and Y)

Ln­(OTf)_3_ was synthesized according to the following modified literature procedure.^70^

The synthesis was performed under aerobic conditions in a well ventilated fumehood. In a 100 mL round-bottomed flask cooled in an ice bath, anhydrous trifluoromethanesulfonic acid (2 mL) was added to miliQ water (2 mL) (*Caution! Exothermic reaction*). The reaction was stirred until fuming stopped. Afterward, Ln_2_O_3_ (5.73 mmol) was added portion-wise under strong stirring, and the suspension was heated at 110 °C for 2 h. The reaction was allowed to cool down to room temperature and diluted with water (50 mL). The mixture was filtered to remove the unreacted oxide and the solution was further filtered using Acrodisc syringe filters (0.2 mm, 13 mm) resulted in a clear solution which was dried under vacuum to afford a white powder. The triflate was dried under vacuum (10^–5^ bar) at 200 °C for 24 h prior use.

The triflate was dried under vacuum (10^–5^ bar) at 200 °C for 24 h prior use. All the obtained triflates appear as white solids apart from Pr­(OTf)_3_ which is pale green, Nd­(OTf)_3_ which is purple, and Er­(OTf)_3_ which is light pink.

#### Synthesis of [NEt_4_]_3_[CeCl_2_(MeCN)­(WS_4_)_2_] (**1Ce**)

In a 5 mL scintillation vial, (NEt_4_)_2_WS_4_ (50 mg, 0.087 mmol, 2 equiv) and NEt_4_Cl (14.5 mg, 0.087 mmol, 2 equiv) were solubilized in 2 mL of MeCN resulting in a bright yellow solution. The latter was treated with Ce­(OTf)_3_ (25.6 mg, 0.043 mmol, 1 equiv) resulting in an immediate color change to dark orange. The reaction was stirred at room temperature for 24 h, before the solution set to crystallize at −35 °C by vapor diffusion, using a 25 mL scintillation vial filled with a Et_2_O (3 mL) (49.8 mg, 39 mmol, 91% yield). Elem. Anal. Found (calcd), %, for C_26_H_63_CeCl_2_N_4_S_8_W_2_: C, 24.58 (24.65); H, 5.00 (5.01); N, 4.37 (4.42)

#### Synthesis of [NEt_4_]_3_[PrCl_2_(MeCN)­(WS_4_)_2_] (**1Pr**)

In a 5 mL scintillation vial, (NEt_4_)_2_WS_4_ (50 mg, 0.087 mmol, 2 equiv) and NEt_4_Cl (14.5 mg, 0.087 mmol, 2 equiv) were solubilized in 2 mL of MeCN resulting in a bright yellow solution. The latter was treated with Pr­(OTf)_3_ (25.6 mg, 0.043 mmol, 1 equiv) resulting in an immediate color change to light orange. The reaction was stirred at room temperature for 24 h, before the solution set to crystallize at −35 °C by vapor diffusion, using a 25 mL scintillation vial filled with Et_2_O (3 mL). Yellow crystals suitable for XRD were collected by pipetting off the supernatant, washed with Et_2_O (2 × 1 mL) and dried under vacuum (46.8 mg, 37 mmol, 86% yield). Elem. Anal. Found (calcd), %, for **1Pr**·0.5Et_2_O C_28_H_68_Cl_2_PrN_4_O_0.5_S_8_W_2_: C, 25.95 (25.77); H, 5.46 (5.25); N, 4.66 (4.29)

#### Synthesis of [NEt_4_]_3_[NdCl_2_(MeCN)­(WS_4_)_2_] (**1Nd**)

In a 5 mL scintillation vial, (NEt_4_)_2_WS_4_ (50 mg, 0.087 mmol, 2 equiv) and NEt_4_Cl (14.5 mg, 0.087 mmol, 2 equiv) were solubilized in 2 mL of MeCN resulting in a bright yellow solution. The latter was treated with Nd­(OTf)_3_ (25.8 mg, 0.043 mmol, 1 equiv) resulting in an immediate color change to light orange. The reaction was stirred at room temperature for 24 h, before the solution set to crystallize at −35 °C by vapor diffusion, using a 25 mL scintillation vial filled with Et_2_O (3 mL). Yellow crystals suitable for XRD were collected by pipetting off the supernatant, washed with Et_2_O (2 × 1 mL) and dried under vacuum (44.3 mg, 0.035 mmol, 81% yield). Elem. Anal. Found (calcd), %, for C_26_H_63_Cl_2_N_4_NdS_8_W_2_: C, 24.55 (24.57); H, 5.46 (5.00); N, 4.30 (4.41)

#### Synthesis of [NEt_4_]_3_[SmCl_2_(MeCN)­(WS_4_)_2_] (**1Sm**)

In a 5 mL scintillation vial, (NEt_4_)_2_WS_4_ (50 mg, 0.087 mmol, 2 equiv) and NEt_4_Cl (14.5 mg, 0.087 mmol, 2 equiv) were solubilized in 2 mL of MeCN resulting in a bright yellow solution. The latter was treated with Sm­(OTf)_3_ (26.0 mg, 0.043 mmol, 1 equiv) resulting in an immediate color change to bright orange. The reaction was stirred at room temperature for 24 h, before the solution set to crystallize at −35 °C by vapor diffusion, using a 25 mL scintillation vial filled with Et_2_O (3 mL). Yellow crystals suitable for XRD were collected by pipetting off the supernatant, washed with Et_2_O (2 × 1 mL) and dried under vacuum (49.5 mg, 38 mmol, 90% yield). Elem. Anal. Found (calcd), %, for C_26_H_63_Cl_2_N_4_S_8_SmW_2_: C, 24.58? (24.45); H, 5.12 (4.97); N, 4.72 (4.39)

#### Synthesis of [NEt_4_]_3_[EuCl_2_(MeCN)­(WS_4_)_2_] (**1Eu**)

##### Synthesis via EuCl_3_

In a 5 mL scintillation vial, (NEt_4_)_2_WS_4_ (20 mg, 0.035 mmol, 1 equiv) was solubilized in 2 mL of MeCN resulting in a bright yellow solution. The latter was treated with EuCl_3_ (22.5 mg, 0.087 mmol, 2.5 equiv) resulting in an immediate color change to dark green. The reaction was stirred at room temperature for 30 min, before being filtered and the solution set to crystallize at −35 °C by vapor diffusion, using a 25 mL scintillation vial filled with Et_2_O (3 mL). Red-orange crystals of **1Eu** suitable for single-crystal XRD were collected by pipetting off the supernatant along with colorless hexagonal crystals which were identified as Eu_2_Cl_9_.

##### Synthesis via Eu­(OTf)_3_

In a 5 mL scintillation vial, (NEt_4_)_2_WS_4_ (50 mg, 0.087 mmol, 2 equiv) and NEt_4_Cl (14.5 mg, 0.087 mmol, 2 equiv) were solubilized in 2 mL of MeCN resulting in a bright yellow solution. The latter was cooled at −35 °C for 1h, before it was treated with Eu­(OTf)_3_ (26.1 mg, 0.043 mmol, 1 equiv) resulting in an immediate color change to dark green. The reaction was stirred at room temperature for 30 min, before being filtered and the solution set to crystallize at −35 °C by vapor diffusion, using a 25 mL scintillation vial filled with Et_2_O (3 mL). Red-orange crystals of **1Eu** suitable for XRD were collected by pipetting off the supernatant, washed with Et_2_O (2 × 1 mL), and dried under vacuum (36.4 mg, 0.028 mmol, 66% yield). Elem. Anal. Found (calcd), %, for C_26_H_63_Cl_2_N_4_EuS_8_W_2_: C, 24.79 (24.42); H, 5.37 (4.97); N, 4.71 (4.38)

#### Synthesis of [NEt_4_]_3_[GdCl_2_(MeCN)­(WS_4_)_2_] (**1Gd**)

In a 5 mL scintillation vial, (NEt_4_)_2_WS_4_ (50 mg, 0.087 mmol, 2 equiv) and NEt_4_Cl (14.5 mg, 0.087 mmol, 2 equiv) were solubilized in 2 mL of MeCN resulting in a bright yellow solution. The latter was treated with Gd­(OTf)_3_ (26.4 mg, 0.043 mmol, 1 equiv) resulting in an immediate color change to bright orange. The reaction was stirred at room temperature for 24 h, before the solution set to crystallize at −35 °C by vapor diffusion, using a 25 mL scintillation vial filled with a 1:2 pentane/Et_2_O mixture (3 mL). Orange crystals suitable for XRD were collected by pipetting off the supernatant, washed with Et_2_O (2 × 1 mL), and dried under vacuum (44 mg, 0.034 mmol, 80% yield). Elem. Anal. Found (calcd), %, for **1Gd**·MeCN C_28_H_66_Cl_2_GdN_5_S_8_W_2_: C, 25.38 (25.38); H, 5.19 (5.02); N, 4.83 (5.28)

#### Synthesis of [NEt_4_]_3_[TbCl_2_(MeCN)­(WS_4_)_2_] (**1Tb**)

In a 5 mL scintillation vial, (NEt_4_)_2_WS_4_ (50 mg, 0.087 mmol, 2 equiv) and NEt_4_Cl (14.5 mg, 0.087 mmol, 2 equiv) were solubilized in 2 mL of MeCN resulting in a bright yellow solution. The latter was treated with Tb­(OTf)_3_ (26.5 mg, 0.043 mmol, 1 equiv) resulting in an immediate color change to dark orange-brown. The reaction was stirred at room temperature for 24 h, before the solution set to crystallize at −35 °C by vapor diffusion, using a 25 mL scintillation vial filled with a 1:2 pentane/Et_2_O mixture (3 mL). Orange crystals suitable for XRD were collected by pipetting off the supernatant, washed with Et_2_O (2 × 1 mL), and dried under vacuum (50.4 mg, 0.039 mmol, 91%). Elem. Anal. Found (calcd), %, for **1Tb**·1 MeCN C_28_H_66_Cl_2_N_5_S_8_TbW_2_: C, 25.25 (25.35); H, 5.36 (5.01); N, 5.06 (5.28)

#### Synthesis of [NEt_4_]_3_[DyCl_2_(MeCN)­(WS_4_)_2_] (**1Dy**)

In a 5 mL scintillation vial, (NEt_4_)_2_WS_4_ (56.3 mg, 0.098 mmol, 2 equiv) and NEt_4_Cl (16.3 mg, 0.098 mmol, 2 equiv) were solubilized in 3 mL of MeCN resulting in a bright yellow solution. The latter was treated with Dy­(OTf)_3_ (30 mg, 0.049 mmol, 1 equiv) resulting in an immediate color change to bright orange. The reaction was stirred at room temperature for 24 h, before the solution set to crystallize at −35 °C by vapor diffusion, using a 25 mL scintillation vial filled with a Et_2_O (5 mL). Orange crystals suitable for XRD were collected by pipetting off the supernatant, washed with Et_2_O (2 × 1 mL), and dried under vacuum (53.3 mg, 0.041 mmol, 84% yield). Elem. Anal. Found (calcd), %, for C_26_H_63_Cl_2_DyN_4_S_8_W_2_: C, 24.20 (24.22); H, 4.97 (4.93); N, 4.42 (4.35)

#### Synthesis of [NEt_4_]_3_[HoCl_2_(MeCN)­(WS_4_)_2_] (**1Ho**)

In a 5 mL scintillation vial, (NEt_4_)_2_WS_4_ (50 mg, 0.087 mmol, 2 equiv) and NEt_4_Cl (14.5 mg, 0.087 mmol, 2 equiv) were solubilized in 2 mL of MeCN resulting in a bright yellow solution. The latter was treated with Ho­(OTf)_3_ (26.7 mg, 0.043 mmol, 1 equiv) resulting in an immediate color change to light orange. The reaction was stirred at room temperature for 24 h, before the solution set to crystallize at −35 °C by vapor diffusion, using a 25 mL scintillation vial filled with a 1:2 pentane/Et_2_O mixture (3 mL). Yellow crystals suitable for XRD were collected by pipetting off the supernatant, washed with Et_2_O (2 × 1 mL), and dried under vacuum (43.9 mg, 0.034 mmol, 80% yield). Elem. Anal. Found (calcd), %, for C_26_H_63_Cl_2_N_4_NdS_8_W_2_: C, 24.28 (24.17); H, 4.94 (4.92); N, 4.27 (4.34)

#### Synthesis of [NEt_4_]_3_[ErCl_2_(MeCN)­(WS_4_)_2_] (**1Er**)

In a 5 mL scintillation vial, (NEt_4_)_2_WS_4_ (56 mg, 0.097 mmol, 2 equiv) and NEt_4_Cl (16.2 mg, 0.097 mmol, 2 equiv) were solubilized in 2 mL of MeCN resulting in a bright yellow solution. The latter was treated with Er­(OTf)_3_ (30.0 mg, 0.048 mmol, 1 equiv) resulting in an immediate color change to orange. The reaction was stirred at room temperature for 24 h, before the solution set to crystallize at −35 °C by vapor diffusion, using a 25 mL scintillation vial filled with Et_2_O mixture (3 mL). Yellow crystals suitable for XRD were collected by pipetting off the supernatant, washed with Et_2_O (2 × 1 mL), and dried under vacuum (51.7 mg, 0.040 mmol, 83% yield). Elem. Anal. Found (calcd), %, for C_26_H_63_Cl_2_ErN_4_S_8_W_2_: C, 24.59 (24.13); H, 5.07 (4.91); N, 4.41 (4.33)

#### Synthesis of [NEt_4_]_3_[TmCl_2_(MeCN)­(WS_4_)_2_] (**1Tm**)

In a 5 mL scintillation vial, (NEt_4_)_2_WS_4_ (50 mg, 0.087 mmol, 2 equiv) and NEt_4_Cl (14.5 mg, 0.087 mmol, 2 equiv) were solubilized in 2 mL of MeCN resulting in a bright yellow solution. The latter was treated with Tm­(OTf)_3_ (26.9 mg, 0.043 mmol, 1 equiv) resulting in an immediate color change to bright orange. The reaction was stirred at room temperature for 24 h, before the solution set to crystallize at −35 °C by vapor diffusion, using a 25 mL scintillation vial filled with a 1:1 pentane/Et_2_O mixture (3 mL). Red-orange crystals suitable for XRD were collected by pipetting off the supernatant, washed with Et_2_O (2 × 1 mL), and dried under vacuum (33.6 mg, 0.026 mmol, 60% yield). Elem. Anal. Found (calcd), %, for C_26_H_63_Cl_2_N_4_NdS_8_W_2_: C, 24.25 (24.10); H, 4.99 (4.90); N, 4.24 (4.32)

#### Synthesis of [NEt_4_]_3_[YbCl_2_(MeCN)­(WS_4_)_2_] (**1Yb**)

In a 5 mL scintillation vial, (NEt_4_)_2_WS_4_ (50 mg, 0.087 mmol, 2 equiv) and NEt_4_Cl (14.5 mg, 0.087 mmol, 2 equiv) were solubilized in 2 mL of MeCN resulting in a bright yellow solution. The latter was treated with Yb­(OTf)_3_ (27.0 mg, 0.043 mmol, 1 equiv) resulting in an immediate color change to dark red. The reaction was stirred at room temperature for 24 h, before the solution set to crystallize at −35 °C by vapor diffusion, using a 25 mL scintillation vial filled with a 1:1 pentane/Et_2_O mixture (3 mL). Orange crystals suitable for XRD were collected by pipetting off the supernatant, washed with Et_2_O (2 × 1 mL), and dried under vacuum (43.0 mg, 0.035 mmol, 82% yield). Elem. Anal. Found (calcd), %, for C_26_H_63_Cl_2_N_4_S_8_W_2_Yb: C, 24.08 (24.02); H, 4.95 (4.89); N, 4.37 (4.31)

#### Synthesis of [NEt_4_]_3_[YCl_2_(MeCN)­(WS_4_)_2_] (**1Y**)

In a 5 mL scintillation vial, (NEt_4_)_2_WS_4_ (64 mg, 0.112 mmol, 2 equiv) and NEt_4_Cl (18.5 mg, 0.112 mmol, 2 equiv) were solubilized in 3 mL of MeCN resulting in a bright yellow solution. The latter was treated with Y­(OTf)_3_ (30.0 mg, 0.056 mmol, 1 equiv) resulting in an immediate color change to orange. The reaction was stirred at room temperature for 24 h, before the solution set to crystallize at −35 °C by vapor diffusion, using a 25 mL scintillation vial filled with a Et_2_O (5 mL). Yellow crystals suitable for XRD were collected by pipetting off the supernatant, washed with Et_2_O (2 × 1 mL), and dried under vacuum (56.2 mg, 0.046 mmol, 83% yield). Elem. Anal. Found (calcd), %, for C_26_H_63_Cl_2_N_4_S_8_W_2_Y: C, 25.75 (25.69); H, 5.60 (5.22); N, 4.27 (4.61)

#### Synthesis of [NEt_4_]_6_[La_2_Cl_2_(WS_4_)_5_] (**2La**)

In a 5 mL scintillation vial, (NEt_4_)_2_WS_4_ (50 mg, 0.087 mmol, 2 equiv) and NEt_4_Cl (14.5 mg, 0.087 mmol, 2 equiv) were solubilized in 3 mL of MeCN resulting in a bright yellow solution. The latter was treated with La­(OTf)_3_ (25.6 mg, 0.043 mmol, 1 equiv) and color changed to a darker yellow. The reaction was stirred at room temperature for 24 h, before it was filtered and the filtrate set to crystallize at −35 °C by vapor diffusion, using a 25 mL scintillation vial filled with Et_2_O (3 mL) (52.8 mg, 0.020 mmol, 96% yield). Elem. Anal. Found (calcd), %, for C_52_H_126_Cl_2_La_2_N_8_S_20_W_5_ (**2La**·2 MeCN): C, 22.36 (22.53); H, 4.90 (4.58); N, 3.78 (4.04)

#### Synthesis of [NEt_4_]_3_[LaBr_2_(MeCN)­(WS_4_)_2_] (**3La**)

In a 5 mL scintillation vial, (NEt_4_)_2_WS_4_ (50 mg, 0.087 mmol, 2 equiv) and NEt_4_Br (18.4 mg, 0.087 mmol, 2 equiv) were solubilized in 3 mL of MeCN resulting in a bright yellow solution. The latter was treated with La­(OTf)_3_ (25.5 mg, 0.043 mmol, 1 equiv) resulting in an immediate color change to light orange. The reaction was stirred at room temperature for 24 h, before it was filtered and the filtrate set to crystallize at −35 °C by vapor diffusion, using a 25 mL scintillation vial filled with a Et_2_O (3 mL) (52.2 mg, 0.039 mmol, 91% yield).

## Supplementary Material



## Notes

Single crystal X-ray diffraction, continuous shape measures calculations, raman spectroscopy, buried volume calculations, solid state luminescence measurements, magnetic measurements, and computational details (PDF)
