# Supplementary material for: Seven-Coordinate Lanthanide Bis-Halide Bis-Tetrathiometallate Complexes: A Compelling Platform for Luminescent and Magnetic Properties
Source: Inorg Chem. 2025 Dec 16;65(1):360–71. doi: 10.1021/acs.inorgchem.5c04468 (PMC12801306; doi:10.1021/acs.inorgchem.5c04468)
Supplement: Supplementary file 1 [file ic5c04468_si_001.pdf]

Supporting Information

Seven-Coordinate Lanthanide Bis-Halide Bis-Tetrathiometallate Complexes: A Compelling Platform for Luminescent and Magnetic Properties

Marie A. Perrin,[1] Salauat R. Kiraev,[2] Julia Specht,[1] Liam Grunwald,[1] Fabrice Pointillart,[3] Olivier Cador,[3] Boris LeGuennic,[3] Olivier Maury,[2] Victor Mougel[1]*

[1]Department of Chemistry and Applied Biosciences, ETH Zürich, Vladimir-Prelog-Weg 1-5, 8093 Zürich, Switzerland.

[2]CNRS, ENS de Lyon, LCH, UMR 5182, 69342, Lyon cedex 07, France

[3]Univ Rennes, CNRS, ISCR (Institut des Sciences Chimiques de Rennes), UMR 6226, 35000 Rennes, France.

*Corresponding author. E-mail: mougel@inorg.chem.ethz.ch

Supplementary Information

1. SINGLE CRYSTAL X-RAY DIFFRACTION 5
2. CONTINUOUS SHAPE MEASURES CALCULATIONS 17
3. RAMAN SPECTROSCOPY 22
4. BURIED VOLUME CALCULATIONS 22
5. SOLID STATE LUMINESCENCE MEASUREMENTS 31
6. MAGNETIC MEASUREMENTS 32
7. COMPUTATIONAL DETAILS 37

Index Supplementary Figures:

Figure S1: Mercury diagram of the solid-state molecular structure of **1Ce**. Counterions, hydrogen atoms and co-crystallized MeCN molecules have been omitted for clarity. ORTEP thermal ellipsoids are shown at the 50% probability level. Selected bond lengths and angles are summarized in Table S1. 5
Figure S2: Mercury diagram of the solid-state molecular structure of **1Pr**. Counterions, hydrogen atoms and co-crystallized MeCN molecules have been omitted for clarity. ORTEP thermal ellipsoids are shown at the 50% probability level. Selected bond lengths and angles are summarized in Table S1. 5
Figure S3: Mercury diagram of the solid-state molecular structure of **1Nd**. Counterions, hydrogen atoms and co-crystallized MeCN molecules have been omitted for clarity. ORTEP thermal ellipsoids are shown at the 50% probability level. Selected bond lengths and angles are summarized in Table S1. 5
Figure S4: Mercury diagram of the solid-state molecular structure of **1Sm**. Counterions, hydrogen atoms and co-crystallized MeCN molecules have been omitted for clarity. ORTEP thermal ellipsoids are shown at the 50% probability level. Selected bond lengths and angles are summarized in Table S1. 6
Figure S5: Mercury diagram of the solid-state molecular structure of **1Eu**. Counterions, hydrogen atoms and co-crystallized MeCN molecules have been omitted for clarity. ORTEP thermal ellipsoids are shown at the 50% probability level. Selected bond lengths and angles are summarized in Table S1. 6
Figure S6: Mercury diagram of the solid-state molecular structure of **1Gd**. Counterions, hydrogen atoms and co-crystallized MeCN molecules have been omitted for clarity. ORTEP thermal ellipsoids are shown at the 50% probability level. Selected bond lengths and angles are summarized in Table S1. 6
Figure S7: Mercury diagram of the solid-state molecular structure of **1Tb**. Counterions, hydrogen atoms and co-crystallized MeCN molecules have been omitted for clarity. ORTEP thermal ellipsoids are shown at the 50% probability level. Selected bond lengths and angles are summarized in Table S1. 7
Figure S8: Mercury diagram of the solid-state molecular structure of **1Dy**. Counterions, hydrogen atoms and co-crystallized MeCN molecules have been omitted for clarity. ORTEP thermal ellipsoids are shown at the 50% probability level. Selected bond lengths and angles are summarized in Table S1. 7
Figure S9: Mercury diagram of the solid-state molecular structure of **1Ho**. Counterions, hydrogen atoms and co-crystallized MeCN molecules have been omitted for clarity. ORTEP thermal ellipsoids are shown at the 50% probability level. Selected bond lengths and angles are summarized in Table S2. 7
Figure S10: Mercury diagram of the solid-state molecular structure of **1Er**. Counterions, hydrogen atoms and co-crystallized MeCN molecules have been omitted for clarity. ORTEP thermal ellipsoids are shown at the 50% probability level. Selected bond lengths and angles are summarized in Table S2. 8
Figure S11: Mercury diagram of the solid-state molecular structure of **1Tm**. Counterions, hydrogen atoms and co-crystallized MeCN molecules have been omitted for clarity. ORTEP thermal ellipsoids are shown at the 50% probability level. Selected bond lengths and angles are summarized in Table S2. 8
Figure S12: Mercury diagram of the solid-state molecular structure of **1Yb**. Counterions, hydrogen atoms and co-crystallized MeCN molecules have been omitted for clarity. ORTEP thermal ellipsoids are shown at the 50% probability level. Selected bond lengths and angles are summarized in Table S2. 8
Figure S13: Mercury diagram of the solid-state molecular structure of **1Y**. Counterions, hydrogen atoms and co-crystallized MeCN molecules have been omitted for clarity. ORTEP thermal ellipsoids are shown at the 50% probability level. Selected bond lengths and angles are summarized in Table S2. 9
Figure S14: Mercury diagram of the solid-state molecular structure of **2La**. Counterions, hydrogen atoms and co-crystallized MeCN molecules have been omitted for clarity. ORTEP

thermal ellipsoids are shown at the 50% probability level. Selected bond lengths and angles are summarized in Table S2. 9
Figure S15: Mercury diagram of the solid-state molecular structure of **3La**. Counterions, hydrogen atoms and co-crystallized MeCN molecules have been omitted for clarity. ORTEP thermal ellipsoids are shown at the 50% probability level. Selected bond lengths and angles are summarized in Table S2. 9
Figure S16: Normalized Raman spectra in MeCN of complexes **1Ln** in the ν_{W-S} region. 22
Figure S17: Normalized Raman spectra in MeCN of complexes **1Ln** in the ν_{Ln-Cl} region. 22
Figure S18: Step 1 23
Figure S19: Step 2....24
Figure S20: Step 3 24
Figure S21: Step 4 25
Figure S22: Step 5 25
Figure S23: Step 6 26
Figure S24: Step 7....26
Figure S25: Step 8 27
Figure S26: Step 9 27
Figure S27: Step 10....28
Figure S28: Normalized excitation (λ_{em}=1029 nm, black; λ_{em}=1050 nm blue) and emission (λ_{exc}=360 nm, red) of crystalline **1Yb** samples at 293 K (top) and 77 K (bottom). 31
Figure S29: The NIR emission decay (λ_{exc}=360 nm, λ_{em}=1016 nm, blue) and the monoexponential fit (red) of **1Yb** crystals at 293 K. R^2 (COD)=0.9761, reduced χ^2=6.46868•10^{-9}. 31
Figure S30: Experimental thermal variation of $\chi_M T$ for **1Dy** (open circles) with calculated curve (see text) in full red line. Inset: field variation of the magnetization of **1Dy** at 2 K (open circles) with calculated curve (see text) in full red line. 32
Figure S31: Experimental variation of the in-phase component (χ_M') of the ac susceptibility as a function of the frequency of the oscillating alternating magnetic (3 Oe amplitude) measured in zero external constant field for **1Dy** at various temperatures between 2 and 24 K. 33
Figure S32: Frequency dependence of the in-phase (●) and out-of-phase (○) components of the ac susceptibility measured on powders at 16 K in zero dc field with the best fitted curves (red lines) for compound **1Dy**. Best fitted parameters are reported in Tables here below....33
Figure S33: Experimental variation of the in-phase component (χ_M') of the ac susceptibility as a function of the frequency of the oscillating alternating magnetic (3 Oe amplitude) measured in zero external constant field for **1Dy@Y** at various temperatures between 2 and 24 K. 35
Figure S34: Hysteresis loops for **1Dy** measured at a sweep rate of 16 Oe s^{-1} at various temperatures. 36
Figure S35: Calculated energies of the five first KD of the complex **1Dy**. Colored dashed lines with numbers correspond to the transition moments between electronic states, directly related to the probability the event occurs. 39
Figure S36: DFT-optimized geometries of **3La** (panels a,c) and **1La** (panels b,d), with (c,d) and without (a,b) constraining the respective Ln-N-C angles. 41

1. Single Crystal X-Ray Diffraction

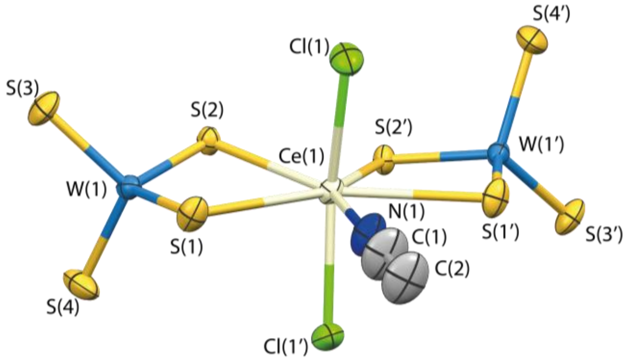

Figure S1: Mercury diagram of the solid-state molecular structure of **1Ce**. Counterions, hydrogen atoms and co-crystallized MeCN molecules have been omitted for clarity. ORTEP thermal ellipsoids are shown at the 50% probability level. Selected bond lengths and angles are summarized in Table S1.

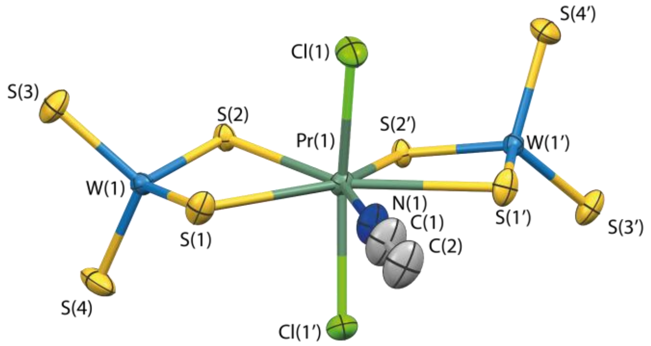

Figure S2: Mercury diagram of the solid-state molecular structure of **1Pr**. Counterions, hydrogen atoms and co-crystallized MeCN molecules have been omitted for clarity. ORTEP thermal ellipsoids are shown at the 50% probability level. Selected bond lengths and angles are summarized in Table S1.

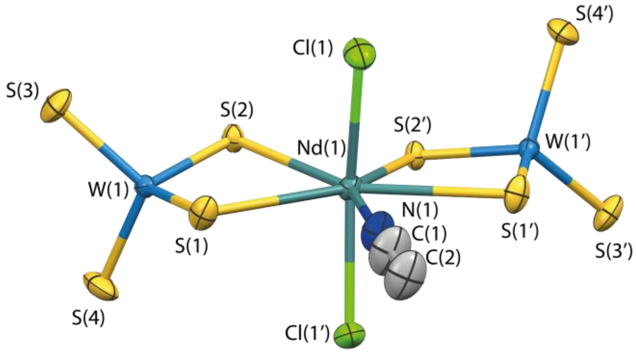

Figure S3: Mercury diagram of the solid-state molecular structure of **1Nd**. Counterions, hydrogen atoms and co-crystallized MeCN molecules have been omitted for clarity. ORTEP thermal ellipsoids are shown at the 50% probability level. Selected bond lengths and angles are summarized in Table S1.

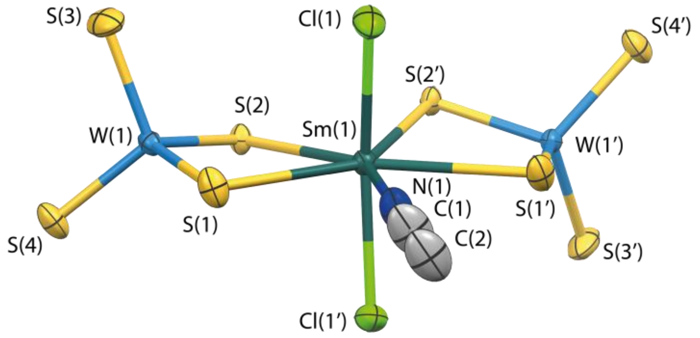

Figure S4: Mercury diagram of the solid-state molecular structure of **1Sm**. Counterions, hydrogen atoms and co-crystallized MeCN molecules have been omitted for clarity. ORTEP thermal ellipsoids are shown at the 50% probability level. Selected bond lengths and angles are summarized in Table S1.

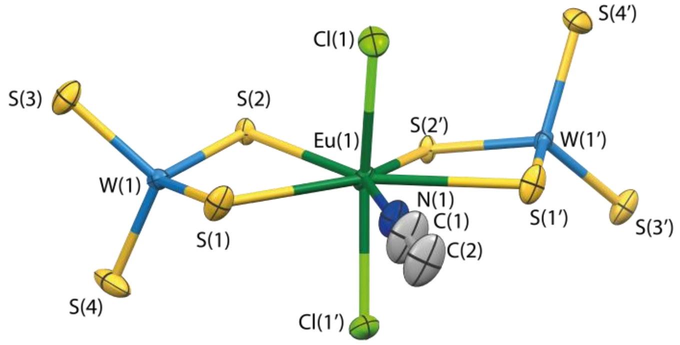

Figure S5: Mercury diagram of the solid-state molecular structure of **1Eu**. Counterions, hydrogen atoms and co-crystallized MeCN molecules have been omitted for clarity. ORTEP thermal ellipsoids are shown at the 50% probability level. Selected bond lengths and angles are summarized in Table S1.

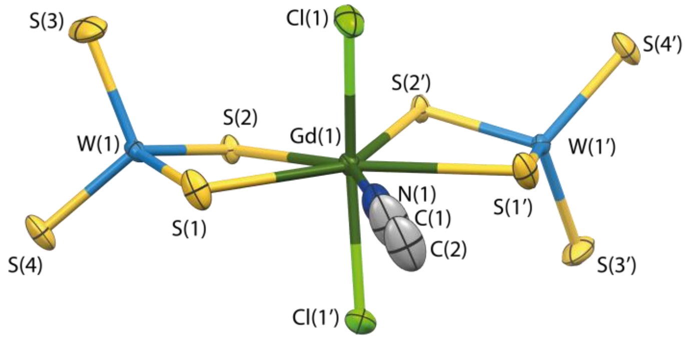

Figure S6: Mercury diagram of the solid-state molecular structure of **1Gd**. Counterions, hydrogen atoms and co-crystallized MeCN molecules have been omitted for clarity. ORTEP thermal ellipsoids are shown at the 50% probability level. Selected bond lengths and angles are summarized in Table S1.

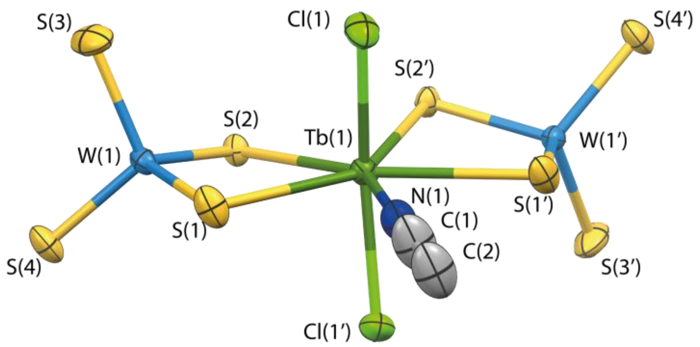

Figure S7: Mercury diagram of the solid-state molecular structure of **1Tb**. Counterions, hydrogen atoms and co-crystallized MeCN molecules have been omitted for clarity. ORTEP thermal ellipsoids are shown at the 50% probability level. Selected bond lengths and angles are summarized in Table S1.

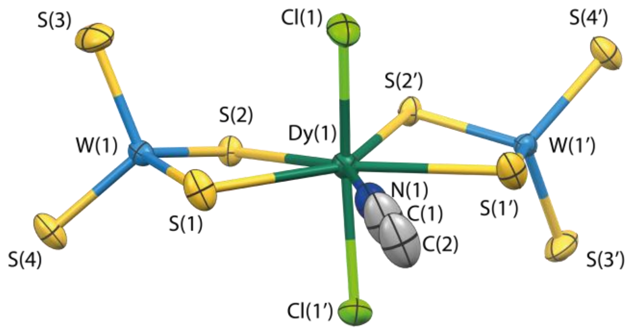

Figure S8: Mercury diagram of the solid-state molecular structure of **1Dy**. Counterions, hydrogen atoms and co-crystallized MeCN molecules have been omitted for clarity. ORTEP thermal ellipsoids are shown at the 50% probability level. Selected bond lengths and angles are summarized in Table S1.

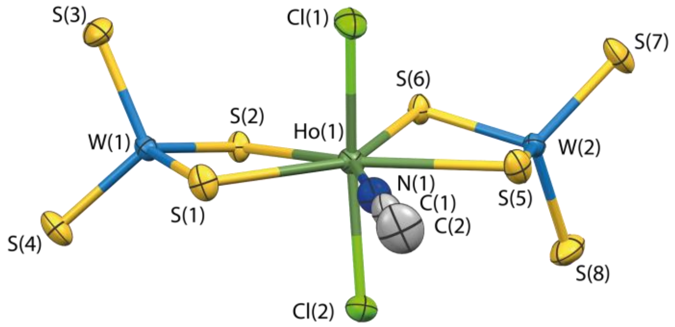

Figure S9: Mercury diagram of the solid-state molecular structure of **1Ho**. Counterions, hydrogen atoms and co-crystallized MeCN molecules have been omitted for clarity. ORTEP thermal ellipsoids are shown at the 50% probability level. Selected bond lengths and angles are summarized in Table S2.

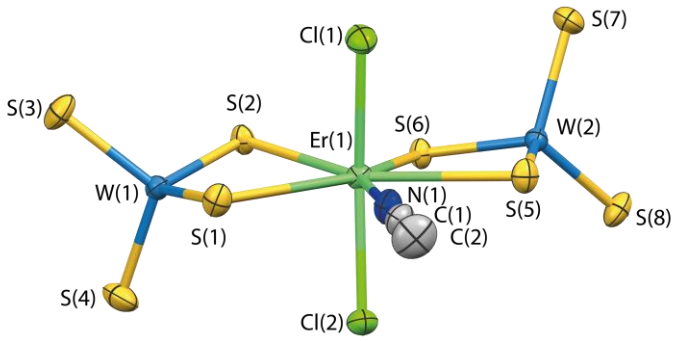

Figure S10: Mercury diagram of the solid-state molecular structure of **1Er**. Counterions, hydrogen atoms and co-crystallized MeCN molecules have been omitted for clarity. ORTEP thermal ellipsoids are shown at the 50% probability level. Selected bond lengths and angles are summarized in Table S2.

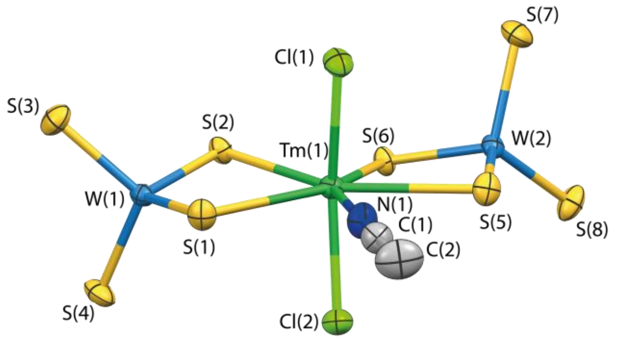

Figure S11: Mercury diagram of the solid-state molecular structure of **1Tm**. Counterions, hydrogen atoms and co-crystallized MeCN molecules have been omitted for clarity. ORTEP thermal ellipsoids are shown at the 50% probability level. Selected bond lengths and angles are summarized in Table S2.

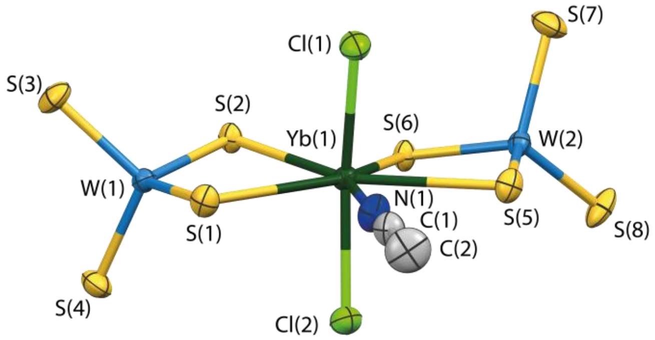

Figure S12: Mercury diagram of the solid-state molecular structure of **1Yb**. Counterions, hydrogen atoms and co-crystallized MeCN molecules have been omitted for clarity. ORTEP thermal ellipsoids are shown at the 50% probability level. Selected bond lengths and angles are summarized in Table S2.

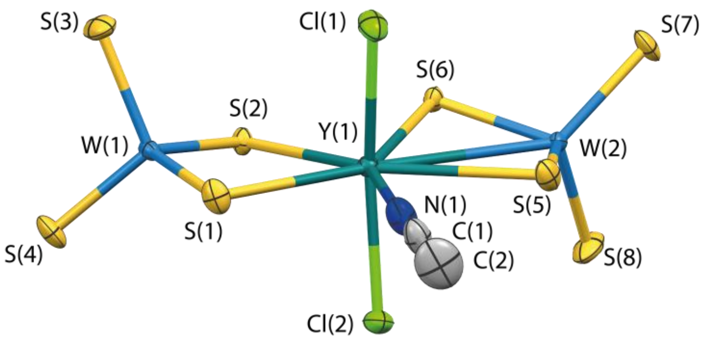

Figure S13: Mercury diagram of the solid-state molecular structure of **1Y**. Counterions, hydrogen atoms and co-crystallized MeCN molecules have been omitted for clarity. ORTEP thermal ellipsoids are shown at the 50% probability level. Selected bond lengths and angles are summarized in Table S2.

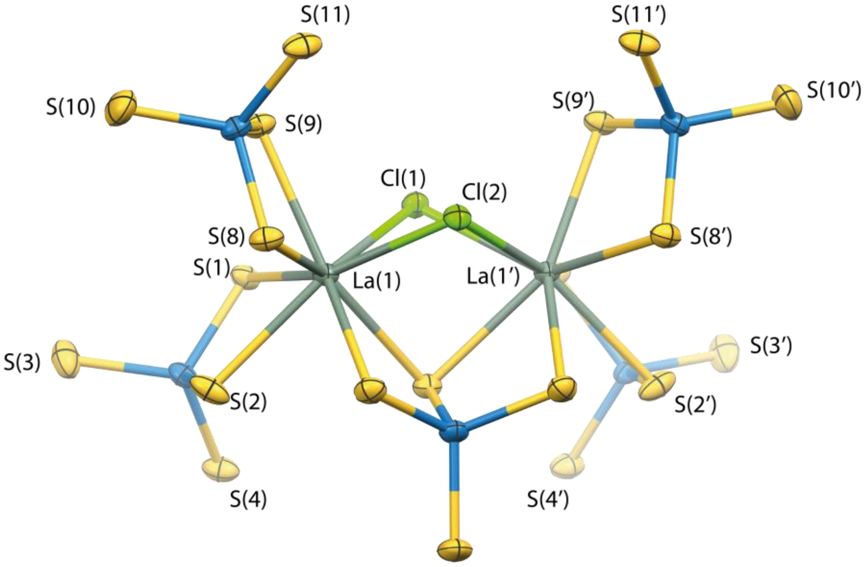

Figure S14: Mercury diagram of the solid-state molecular structure of **2La**. Counterions, hydrogen atoms and co-crystallized MeCN molecules have been omitted for clarity. ORTEP thermal ellipsoids are shown at the 50% probability level. Selected bond lengths and angles are summarized in Table S2.

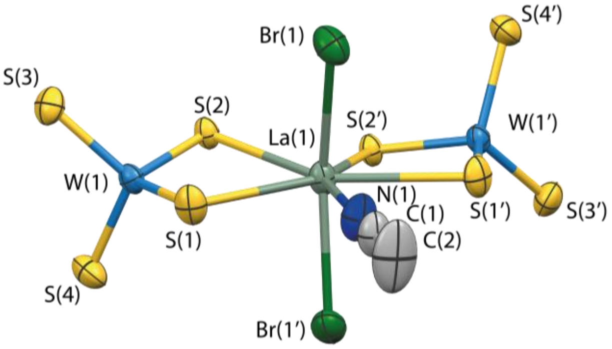

Figure S15: Mercury diagram of the solid-state molecular structure of **3La**. Counterions, hydrogen atoms and co-crystallized MeCN molecules have been omitted for clarity. ORTEP thermal ellipsoids are shown at the 50% probability level. Selected bond lengths and angles are summarized in Table S2.

Table S1 Selected bond distances (Å) and angles (°) for complexes **1Ln** and buried volume calculations (see section 6 for more details on the calculation).

Structural parameters	**1Ce**	**1Pr**	**1Nd**	**1Sm**	**1Eu**	**1Gd**	**1Tb**	**1Dy**
Ln1-W1	3.704	3.6875(2)	3.6734(2)	3.648	3.6366(4)	3.6233(4)	3.6115(4)	3.6069(5)
Ln1-Cl1	2.7131(18)	2.6850(13)	2.6747(12)	2.6476(18)	2.635(2)	2.625(2)	2.607(2)	2.589(2)
Ln1-N1	2.586(11)	2.569(7)	2.548(7)	2.510(10)	2.490(10)	2.482(12)	2.455(11)	2.443(14)
Ln1-S1	3.0109(18)	2.9989(13)	2.9862(12)	2.9692(18)	2.962(2)	2.950(2)	2.945(2)	2.942(3)
Ln1-S2	2.9391(16)	2.9244(12)	2.9063(11)	2.8788(16)	2.8702(17)	2.859(2)	2.8452(19)	2.841(2)
W1-S1	2.2000(18)	2.1974(13)	2.1971(12)	2.1977(18)	2.1971(19)	2.198(2)	2.195(2)	2.194(2)
W1-S2	2.2203(15)	2.2195(11)	2.2210(10)	2.2211(15)	2.2198(16)	2.2225(19)	2.2204(18)	2.221(2)
W1-S3	2.1712(17)	2.1715(13)	2.1703(12)	2.1732(18)	2.168(2)	2.170(2)	2.171(2)	2.174(2)
W1-S4	2.175(2)	2.1757(14)	2.1750(13)	2.1778(19)	2.176(2)	2.179(2)	2.178(2)	2.181(3)
N1-C1	1.138(17)	1.117(12)	1.115(11)	1.129(16)	1.142(17)	1.13(2)	1.132(18)	1.12(2)
C1-C2	1.417(17)	1.430(12)	1.425(12)	1.420(16)	1.425(17)	1.41(2)	1.420(19)	1.42(2)
Cl1-Ln1-Cl1’	162.23(9)	162.77(7)	162.78(6)	163.00(9)	163.35(10)	163.45(12)	163.86(11)	164.53(13)
S1-Ln1-S1’	145.29(7)	144.75(5)	144.17(5)	143.00(7)	142.65(8)	142.25(9)	141.77(8)	141.59(10)
CShM**	1.122	1.099	1.099	1.107	1.109	1.098	1.113	1.114
%Vbur 1 (R=3 Å)	65.2	66.2	67.0	68.5	69.1	69.6	70.4	71
%Vbur 2 (R=5 Å)	41.9	42.0	42.2	42.5	42.5	42.7	42.7	42.9
Delta %Vbur	23.3	24.2	24.8	26	26.6	26.9	27.7	28.8

*averaged from Ln–W1 and Ln–W2 bonds and Ln–Cl1 and Ln–Cl2 bonds. **CShM=continuous shape measures for PB geometry

Table S2: Selected bond distances (Å) and angles (°) for complexes **1XLn** and **2La** and buried volume calculations (see section 6 for more details on the calculation).

Structural parameters	**1Ho***	**1Er***	**1Tm***	**1Yb***	**1Y***	**3La**	Structural parameters	**2La**
Ln1-W1	3.595(0)	3.585(7)	3.5790(2)	3.573	3.6042(8)	3.7245(4)	La1-Cl1	2.9178(10)
Ln1-X1	2.5852	2.574(5)	2.559(7)	2.541(7)	2.5907	2.9051(11)	La1-Cl2	2.8580(10)
Ln1-N1	2.433(8)	2.418(6)	2.392(7)	2.387(6)	2.429(5)	2.635(12)	La1-La1’	4.1502(5)
Ln1-S1	2.940(2)	2.9361(18)	2.923(2)	2.9196(19)	2.948(2)	3.016(2)	La1-S1	2.9597(10)
Ln1-S2	2.821(2)	2.8131(17)	2.808(2)	2.8022(17)	2.8346(18)	2.9608(18)	La1-S2	2.9921(11)
W1-S1	2.193(2)	2.1926(17)	2.197(2)	2.1968(18)	2.1895(17)	2.203(2)	La1-S5	3.0271(11)
W1-S2	2.2196(19)	2.2205(16)	2.2211(19)	2.2197(17)	2.2220(16)	2.2225(17)	La1-S7	3.1132(11)
W1-S3	2.173(2)	2.1735(19)	2.167(2)	2.1700(18)	2.1889(18)	2.181(2)	La1-S8	2.9508(11)
W1-S4	2.183(2)	2.1809(18)	2.173(2)	2.181(2)	2.1729(19)	2.172(2)	La1-S9	2.9833(10)
N1-C1	1.132(12)	1.123(9)	1.140(11)	1.138(10)	1.133(8)	1.112(19)	W2-S5	2.2323(15)
C1-C2	1.439(13)	1.438(10)	1.454(12)	1.423(11)	1.441(9)	1.440(18)	W2-S6	2.1630(16)
X1-Ln1-X1’	164.57(8)	164.39(6)	164.64(6)	165.12(6)	164.47(5)	158.54(5)	W2-S7	2.1894(12)
S1-Ln1-S1’	141.18(6)	140.76(4)	140.48(5)	140.27(5)	141.53(4)	147.32(8)		
CShM**	1.098	1.126	1.162	1.150	1.124	1.443		
%Vbur 1 (R=3 Å)	71.5	72	72.5	73	71.1	63.9	%Vbur 1 (R=3 Å)	71.6
%Vbur 2 (R=5 Å)	42.9	43	43.1	43.1	42.9	43.3	%Vbur 2 (R=5 Å)	55.4
Delta %Vbur	28.6	29	29.4	29.9	28.2	20.6	Delta %Vbur	16.2

*averaged from Ln–W1 and Ln–W2 bonds and Ln–Cl1 and Ln–Cl2 bonds. **CShM=continuous shape measures for PB geometry

Table S3: Refinement parameters for complexes **1Ce**, **1Pr** and **1Nd**.

	**1Ce**	**1Pr**	**1Nd**
Formula	$[NEt_4]_3[Ce^{III}(MeCN)Cl_2(WS_4)_2]$	$[NEt_4]_3[Pr^{III}(MeCN)Cl_2(WS_4)_2]$	$[NEt_4]_3[Nd(MeCN)Cl_2(WS_4)_2]$
Empirical formula	$C_{26}H_{63}CeCl_2N_4S_8W_2$	$C_{26}H_{63}Cl_2N_4PrS_8W_2$	$C_{26}H_{63}Cl_2N_4NdS_8W_2$
Molecular weight [g/mol]	1267.00	1267.79	1271.12
Temperature [K]	100	100	100
Crystal system	*monoclinic*	*monoclinic*	*monoclinic*
Space group	*C2/c*	*C2/c*	*C2/c*
a [å]	12.3522(2)	12.32510(10)	12.30388(6)
b [å]	30.8234(5)	30.7636(2)	30.67986(13)
c [å]	11.7223(2)	11.69990(10)	11.69808(5)
α [°]	90	90	90
β [°]	95.290(2)	95.1340(10)	95.2019(4)
γ [°]	90	90	90
V [$Å^3$]	4444.10(13)	4418.39(6)	4397.62(4)
Z	4	4	4
ρ_{calc} [$g{\cdot}cm^{-3}$]	1.894	1.906	1.920
μ [mm^{-1}]	21.927	22.552	23.168
F(000)	2460.0	2464.0	2468.0
Crystal size [mm^3]	0.073 × 0.059 × 0.014	0.159 × 0.033 ×0.031	0.226 × 0.217 × 0.12
Radiation	CuKα (λ=1.54184)	CuKα (λ=1.54184)	CuKα (λ=1.54184)
2θ range [°]	5.734 to 160.24	5.746 to 160.2	5.762 to 160.152
Reflections collected	18076	45508	93560
Independent reflexions	4706	4802	4792
Final r indexes [i>=2σ (i)]	R1=0.0450, wR2=0.1112	R1=0.0330 wR2=0.0850	R1=0.0297, wR2=0.0699
Final r indexes [all data]	R1=0.0504, wR2=0.1141	R1=0.0334 wR2=0.0853	R1=0.0299 wR2=0.0700
Goof F^2	1.056	1.110	1.143
Largest diff. Peak/hole [$e{\cdot}Å^{-3}$]	1.86/-1.23	1.57/-1.19	1.67/-1.36
CSD number	2376301	2376302	2376303

Table S4: Refinement parameters for complexes **1Sm, 1Eu** and **1Gd**

	**1Sm**	**1Eu**	**1Gd**
Formula	$[NEt_4]_3[Sm(MeCN)Cl_2(WS_4)_2]$	$[NEt_4]_3[Eu(MeCN)Cl_2(WS_4)_2]$	$[NEt_4]_3[Gd(MeCN)Cl_2(WS_4)_2]$
Empirical formula	$C_{26}H_{63}Cl_2N_4S_8SmW_2$	$C_{26}H_{63}Cl_2EuN_4S_8W_2$	$C_{26}H_{63}Cl_2GdN_4S_8W_2$
Molecular weight [g/mol]	1277.24	1278.84	1284.13
Temperature [K]	100	100	100
Crystal system	*monoclinic*	*monoclinic*	*monoclinic*
Space group	*C2/c*	*C2/c*	*C2/c*
a [å]	12.31020(10)	12.30090(5)	12.26169(5)
b [å]	30.6582(3)	30.62353(11)	30.5849113
c [å]	11.70380(10)	11.69755(5)	11.68641(5)
α [°]	90	90	90
β [°]	95.3340(10)	95.3352(4)	95.3747(4)
γ [°]	90	90	90
V [$Å^3$]	4397.99(7)	4387.34(3)	4363.40(3)
Z	4	4	4
ρ_{calc} [$g \cdot cm^{-3}$]	1.890	1.936	1.955
μ [mm^{-1}]	24.176	24.426	24.101
F(000)	2396.0	2480.0	2484.0
Crystal size [mm^3]	0.105× 0.075 × 0.028	0.147 × 0.103 ×0.032	0.152 × 0.136 × 0.110
Radiation	CuKα (λ=1.54184)	CuKα (λ=1.54184)	CuKα (λ=1.54184)
2θ range [°]	5.766 to 160.754	5.772 to 160.518	5.78 to 160.554
Reflections collected	37117	108488	105210
Independent reflexions	4736	4789	4758
Final r indexes [i>=2σ (i)]	R1=0.0416, wR2=0.0993	R1=0.0430, wR2=0.1029	R1=0.0478, wR2=0.1146
Final r indexes [all data]	R1=0.0423, wR2=0.10996	R1=0.0431 wR2=0.1029	R1=0.0478, wR2=0.1146
Goof F^2	1.181	1.243	1.267
Largest diff. Peak/hole [$e \cdot Å^{-3}$]	2.45/-1.79	1.36/-1.39	1.60/-2.28
CSD number	2376304	2376305	2376306

Table S5: Refinement parameters for complexes **1Tb, 1Dy** and **1Ho**

	**1Tb**	**1Dy**	**1Ho**
Formula	$[NEt_4]_3[Tb^{III}(MeCN)Cl_2(WS_4)_2]$	$[NEt_4]_3[Dy^{III}(MeCN)Cl_2(WS_4)_2]$	$[NEt_4]_3[Ho^{III}(MeCN)Cl_2(WS_4)_2]$
Empirical formula	$C_{26}H_{63}Cl_2N_4S_8TbW_2$	$C_{26}H_{63}Cl_2DyN_4S_8W_2$	$C_{26}H_{63}Cl_2HoN_4S_8W_2$
Molecular weight [g/mol]	1285.80	1289.38	1291.81
Temperature [K]	100	100	100
Crystal system	*monoclinic*	*monoclinic*	*triclinic*
Space group	*C2/c*	*C2/c*	*P*-1
a [å]	12.26480(5)	12.27630(10)	11.69278(5)
b [å]	30.53701(13)	30.5226(3)	12.24852(6)
c [å]	11.68804(6)	11.68490(10)	16.42590(8)
α [°]	90	90	68.1712(5)
β [°]	95.4312(4)	95.4320(10)	87.9093(4)
γ [°]	90	90	84.4920(4)
V [Å^3]	4357.88(3)	4358.72	2173.75(2)
Z	4	4	2
ρ_{calc} [g·cm^{-3}]	1.960	1.965	1.974
μ [mm^{-1}]	22.269	23.457	17.722
F(000)	2488.0	2492.0	1248.0
Crystal size [mm^3]	0.101× 0.097 × 0.048	0.140 × 0.050 ×0.022	0.193 × 0.067 × 0.033
Radiation	CuKα (λ=1.54184)	CuKα (λ=1.54184)	CuKα (λ=1.54184)
2θ range [°]	5 5.788 to 160.65	5.792 to 160.758	5.796 to 160.576
Reflections collected	102284	27176	109449
Independent reflexions	4743	4701	9362
Final r indexes [i>=2σ (i)]	R1=0.0447, wR2=0.1070	R1=0.0543 wR2=0.1294	R1=0.0500, wR2=0.1218
Final r indexes [all data]	R1=0.0449, wR2=0.1071	R1=0.0550 wR2=0.1297	R1=0.0504, wR2=0.1220
Goof F^2	1.255	1.263	1.264
Largest diff. Peak/hole [e·Å^{-3}]	1.40/-1.74	1.41/-2.18	1.92/-1.44
CSD number	2376307	2376315	2376308

Table S6: Refinement parameters for complexes **1Er**, **1Tm** and **1Yb**

	**1Er**	**1Tm**	**1Yb**
Formula	$[NEt_4]_3[Er^{III}(MeCN)Cl_2(WS_4)_2]$	$[NEt_4]_3[Tm^{III}(MeCN)Cl_2(WS_4)_2]$	$[NEt_4]_3[Yb^{III}(MeCN)Cl_2(WS_4)_2]$
Empirical formula	$C_{26}H_{63}Cl_2ErN_4S_8W_2$	$C_{26}H_{63}Cl_2N_4S_8TmW_2$	$C_{26}H_{63}Cl_2N_4S_8W_2Yb$
Molecular weight [g/mol]	1294.14	1295.81	1299.972
Temperature [K]	100	100	100
Crystal system	*triclinic*	*triclinic*	*triclinic*
Space group	*P*-1	*P*-1	*P*-1
a [å]	11.67970(10)	11.67006(15)	11.65354(14)
b [å]	12.23650(10)	12.2258(2)	12.1921(2)
c [å]	16.4341(2)	16.4096(3)	16.3932(2)
α [°]	68.1510(10)	68.1598(15)	68.3131(14)
β [°]	87.9550(10)	87.9709(12)	87.907(1)
γ [°]	84.5470(10)	84.4863(12)	84.5978(12)
V [Å^3]	2170.16(4)	2163.16(6)	2154.69(6)
Z	2	2	2
ρ_{calc} [g·cm^{-3}]	1.980	1.989	2.004
μ [mm^{-1}]	17.935	18.234	18.462
F(000)	1250.0	1252.0	1228.6
Crystal size [mm^3]	0.119× 0.103 × 0.092	0.117 × 0.078 × 0.016	0.137 × 0.098 × 0.083
Radiation	CuKα (λ=1.54184)	CuKα (λ=1.54184)	CuKα (λ=1.54184)
2θ range [°]	5.794 to 160.656	5.802 to 160.044	5.8 to 160.48
Reflections collected	49054	25214	26878
Independent reflexions	9231	8847	8766
Final r indexes [i>=2σ (i)]	R1=0.0339, wR2=0.0895	R1=0.0382 wR2=0.1017	R1=0.0409, wR2=0.0992
Final r indexes [all data]	R1=0.0341 wR2=0.0897	R1=0.0392 wR2=0.1024	R1=0.0416, wR2=0.0998
Goof F^2	1.095	1.111	1.030
Largest diff. Peak/hole [e·Å^{-3}]	1.71/-2.27	1.33/-1.64	3.43/-3.60
CSD number	2376309	2376310	2376311

Table S7: Refinement parameters for complexes **1Y**, **2La** and **3La**

	**1Y**	**2La**	**3La**
Formula	$[NEt_4]_3[Y^{III}(MeCN)Cl_2(WS_4)2]$	$[NEt_4]_6[La_2^{III}Cl_2(WS_4)_5]$	$[NEt_4]_3[La^{III}(MeCN)Br_2(WS_4)2]$
Empirical formula	$C_{26}H_{63}Cl_2N_4S_8W_2Y$	$C_{48}H_{120}Cl_2La_2N_6S_{20}W_5$	$C_{26}H_{63}Br_2LaN_4S_8W_2$
Molecular weight [g/mol]	1215.79	2690.66	1350.68
Temperature [K]	100	100	100
Crystal system	*triclinic*	*orthorhombic*	*orthorhombic*
Space group	*P*-1	*Pnma*	*Fddd*
a [å]	11.68268(10)	42.30897(11)	16.2960(3)
b [å]	12.25976(10)	18.95171(6)	18.0286(3)
c [å]	16.44979(13)	12.40292(3)	62.2955(13)
α [°]	68.1529(8)	90	90
β [°]	87.9758(7)	90	90
γ [°]	84.5965(7)	90	90
V [Å^3]	2177.12(3)	9945.00(5)	18302.1(6)
Z	2	4	16
ρ_{calc} [g·cm^{-3}]	1.855	1.710	1.961
μ [mm^{-1}]	16.187	21.495	21.797
F(000)	1192.0	5152.0	10336.0
Crystal size [mm^3]	0.155 × 1.117 × 0.054	0.173 × 0.037 × 0.015	0.068 × 0.033 × 0.016
Radiation	CuKα (λ=1.54184)	CuKα (λ=1.54184)	CuKα (λ=1.54184)
2θ range [°]	5.788 to 160.358	6.262 to 160.62	5.674 to 162.65
Reflections collected	49886	497886	56802
Independent reflexions	9117	11194	5010
Final r indexes [i>=2σ (i)]	R1=0.0305, wR2=0.0748	R1=0.0333, wR2=0.0887	R1=0.0527, wR2=0.1467
Final r indexes [all data]	R1=0.0308, wR2=0.0750	R1=0.0343 wR2=0.0895	R1=0.0538, wR2=0.1476
Goof F^2	1.049	1.066	1.123
Largest diff. Peak/hole [e·Å^{-3}]	1.02/-1.67	1.20/-1.53	1.42/-2.46
CSD number	2376312	2376313	2376314

2. Continuous Shape Measures Calculations

Imput file (.dat):

```
$ LnCl2W2 structures
! Ligands  Metal
   7    1
! Tetrahedron Square
   1    2    3    4    5    6    7

CECLW
Ce     5.365531   20.386412    8.754279
Cl     7.216399   20.805333    6.815335
Cl     3.514663   20.805333   10.693224
S      4.302367   17.985824    7.433142
S      3.226298   21.284637    6.835295
S      6.428695   17.985824   10.075417
S      7.504765   21.284637   10.673264
N      5.365531   22.972403    8.754279
PRCLW
Pr     5.377323   20.351506    8.739721
Cl     7.212928   20.753740    6.821900
Cl     3.541718   20.753740   10.657542
S      4.317550   17.960374    7.431700
S      3.246223   21.259463    6.835150
S      6.437096   17.960374   10.047743
S      7.508423   21.259463   10.644293
N      5.377323   22.920328    8.739721
NDCLW
Nd    -0.795460    4.957252    8.737425
Cl    -2.627646    5.357624   10.644514
Cl     1.036725    5.357624    6.830336
S      0.253701    2.578949   10.037438
S      1.318069    5.875807   10.636592
S     -1.844621    2.578949    7.437413
S     -2.908989    5.875807    6.838258
N     -0.795460    7.504294    8.737425
```

SMCLW

Sm	12.038199	10.390279	2.913280
Cl	13.849524	9.998835	1.022282
Cl	10.226875	9.998835	4.804278
S	11.001905	12.746085	1.623629
S	9.946292	9.447968	1.028481
S	13.074494	12.746085	4.202930
S	14.130107	9.447968	4.798079
N	12.038199	7.879280	2.913280

EUCLW

Eu	5.334702	20.245980	8.735154
Cl	3.528562	20.627641	10.615704
Cl	7.140843	20.627641	6.854603
S	3.250854	21.194605	6.855663
S	4.303455	17.895471	7.451028
S	6.365949	17.895471	10.019279
S	7.418551	21.194605	10.614644
N	5.334702	22.736408	8.735154

GDCLW

Gd	11.440703	10.357946	8.726273
Cl	13.244193	9.980069	6.856396
Cl	9.637213	9.980069	10.596151
S	9.376873	9.403605	6.846564
S	10.420476	12.704879	7.450794
S	12.460930	12.704879	10.001752
S	13.504532	9.403605	10.605982
N	11.440703	7.875491	8.726273

TBCLW

Tb	11.435095	10.345237	8.726678
Cl	9.645250	9.979220	10.586763
Cl	13.224941	9.979220	6.866592
S	10.420190	12.678967	7.454293
S	9.377265	9.381000	6.854317
S	12.450001	12.678967	9.999062
S	13.492926	9.381000	10.599039
N	11.435095	7.890000	8.726678

DYCLW

Dy	11.446693	10.337699	8.724320
Cl	9.665326	9.989314	10.569048
Cl	13.228059	9.989314	6.879591
S	10.434740	12.668130	7.453256
S	9.390257	9.369980	6.855547
S	12.458646	12.668130	9.995383
S	13.503128	9.369980	10.593092
N	11.446693	7.896960	8.724320

HOCLW

Ho	9.339921	6.015505	4.920270
Cl	11.320410	7.616933	5.329286
Cl	7.318514	4.421416	5.204221
S	8.193707	7.193406	2.614385
S	10.512825	4.950842	2.585978
S	7.657777	8.189944	5.936650
S	10.987644	3.767075	5.853242
N	9.312613	5.941136	7.351982

ERCLW

Er	3.479076	5.999636	10.338496
Cl	5.444391	7.598164	9.913622
Cl	1.462292	4.414437	10.066387
S	2.339104	7.179981	12.630029
S	4.654499	4.950488	12.669017
S	1.795390	8.153941	9.304194
S	5.123567	3.752377	9.407965
N	3.450573	5.912545	7.921760

TMCLW

Tm	3.234303	3.122789	2.706539
Cl	5.243257	4.698962	2.437240
Cl	1.278974	1.534972	2.293909
S	4.374949	1.952910	4.990699
S	4.921097	0.971788	1.670167
S	2.063240	4.172805	5.022991
S	1.589976	5.367694	1.764147
N	3.258888	3.199133	0.316821

YBCLW

Yb	3.8091	9.1849	2.6960

Cl 1.8689 7.6022 2.2861
Cl 5.7910 10.7643 2.4491
S 2.6436 10.2226 5.0123
S 4.9429 8.0130 4.9750
S 2.1611 11.4242 1.7549
S 5.5032 7.0493 1.6506
N 3.8516 9.2828 0.3115
YClW
Y 4.347959 15.177207 12.559163
Cl 6.330877 16.786876 12.970967
Cl 2.320807 13.584784 12.845827
S 2.658038 17.354544 13.572922
S 3.193985 16.359119 10.246617
S 5.528893 14.107484 10.214767
S 6.003225 12.918540 13.482136
N 4.340022 15.114928 14.987292
LaBrW
La 10.185000 11.267875 26.138943
Br 9.880851 8.429813 25.598031
Br 10.489149 14.105937 25.598031
S 11.865884 11.388901 28.573388
S 13.055443 10.898054 25.290478
S 8.504116 11.146849 28.573388
S 7.314557 11.637696 25.290478
N 10.185000 11.267875 23.504341

Output file (.tab):

```
-------------------------------------------------------------------------------
S H A P E   v2.1        Continuous Shape Measures calculation
(c) 2013  Electronic Structure Group, Universitat de Barcelona
                Contact:  llunell@ub.edu
-------------------------------------------------------------------------------
```

LnCl2W2 structures

HP-7 1 D7h Heptagon
HPY-7 2 C6v Hexagonal pyramid

PBPY-7 3 D5h Pentagonal bipyramid
COC-7 4 C3v Capped octahedron
CTPR-7 5 C2v Capped trigonal prism
JPBPY-7 6 D5h Johnson pentagonal bipyramid J13
JETPY-7 7 C3v Johnson elongated triangular pyramid J7

Structure [ML7]	HP-7	HPY-7	PBPY-7	COC-7	CTPR-7	JPBPY-7	JETPY-7
CECLW	33.869	22.549	1.122	6.058	4.092	3.441	21.915
PRCLW	33.963	22.710	1.099	6.139	4.186	3.341	22.021
NDCLW	33.975	22.804	1.099	6.180	4.220	3.371	22.027
SMCLW	33.958	22.996	1.107	6.267	4.289	3.362	22.017
EUCLW	34.011	23.112	1.109	6.321	4.345	3.354	22.046
GDCLW	33.947	23.153	1.098	6.369	4.377	3.345	22.075
TBCLW	33.979	23.299	1.113	6.426	4.430	3.334	22.056
DYCLW	34.058	23.436	1.114	6.480	4.495	3.261	22.110
HOCLW	34.036	23.359	1.098	6.469	4.548	3.286	22.038
ERCLW	33.971	23.357	1.126	6.478	4.578	3.298	21.995
TMCLW	33.970	23.470	1.162	6.542	4.635	3.306	21.964
YBCLW	34.067	23.546	1.150	6.670	4.735	3.215	22.014
YClW	33.936	23.296	1.124	6.411	4.531	3.303	22.034
LaBrW	33.415	21.922	1.443	5.346	3.334	4.678	21.499

3. Raman Spectroscopy

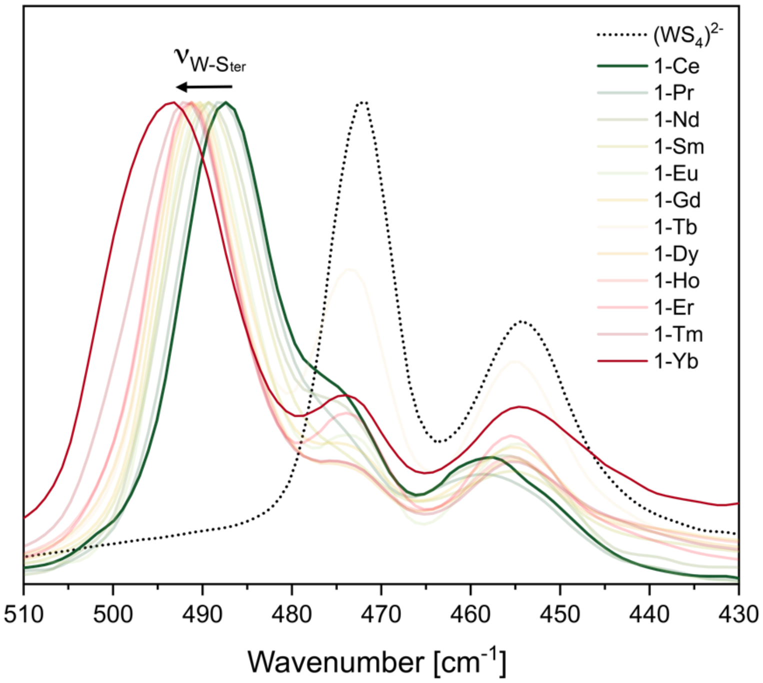

Figure S16: Normalized Raman spectra in MeCN of complexes **1Ln** in the $\nu_{W\text{-}S}$ region.

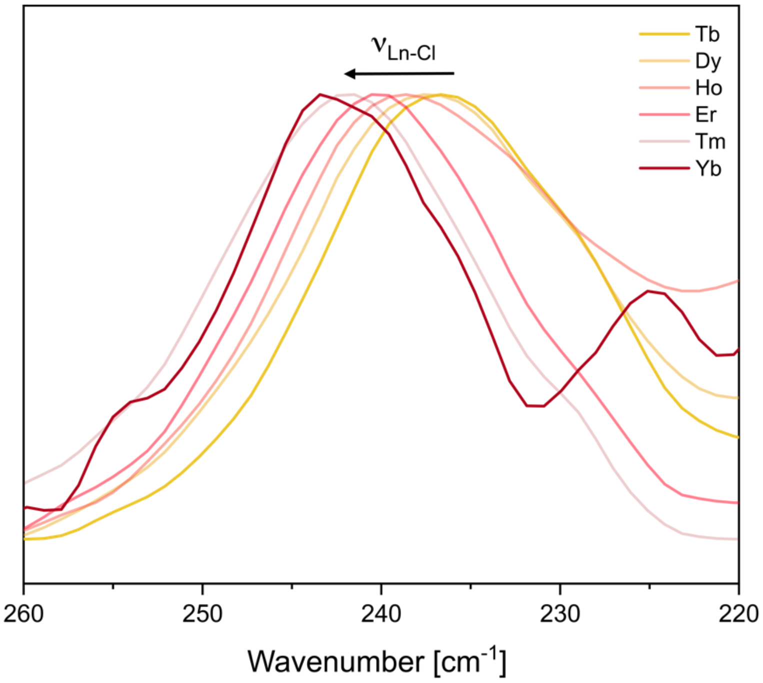

Figure S17: Normalized Raman spectra in MeCN of complexes **1Ln** in the $\nu_{Ln\text{-}Cl}$ region.

4. Buried Volume Calculations

For calculation of the total buried volume, the XYZ input file can be used directly as obtained from single-crystal XRD analysis for the SambVca 2.1 web application. In the application, the following step by step procedure was followed systematically for all complexes.

1. For the center of the sphere, the lanthanide center was selected
2. For the definition of the z axis, the nitrogen atom from the MeCN moiety was selected.
3. For the definition of the xz-plane, select one of the bridging sulfur atoms on the same side as the MeCN moiety.
4. The lanthanide center was then selected to be deleted.
5. The chosen orientation was checked to ensure that the z axis goes along the direction of the MeCN moiety and that the y axis goes along the Ln-Cl bond
6. The Bondi radii was not scaled.
7. The sphere radius was set to 3.0 Å to calculated $\%V_{bur1}$ and 5.0 Å for $\%V_{bur2}$
8. The distance of the coordination point from the center of the sphere was set to 0.0.
9. The mesh spacing for numerical integration was set to 0.1 Å.
10. H atoms were not included in the calculation.

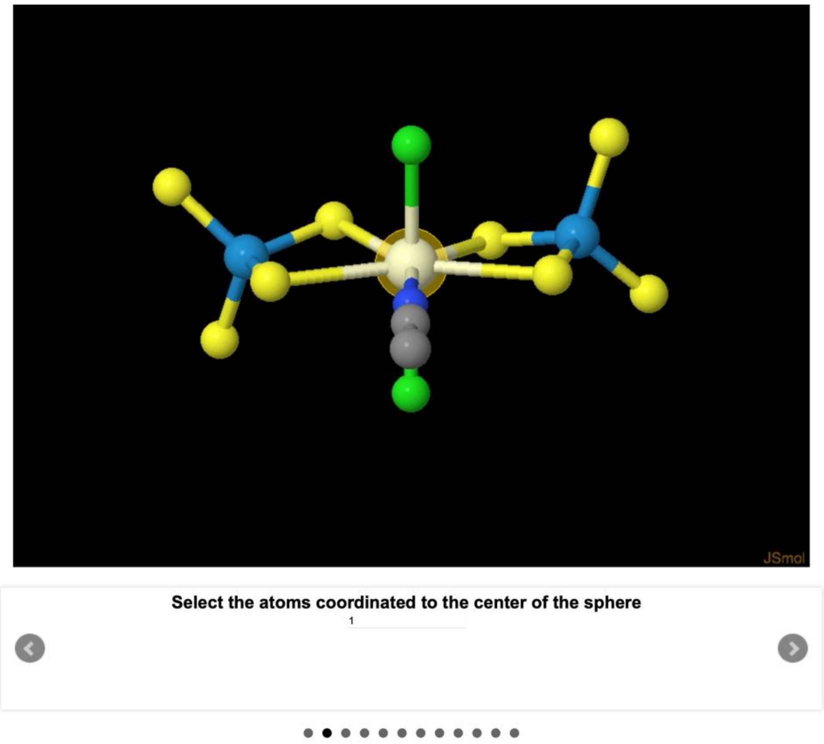

Figure S18: Step 1

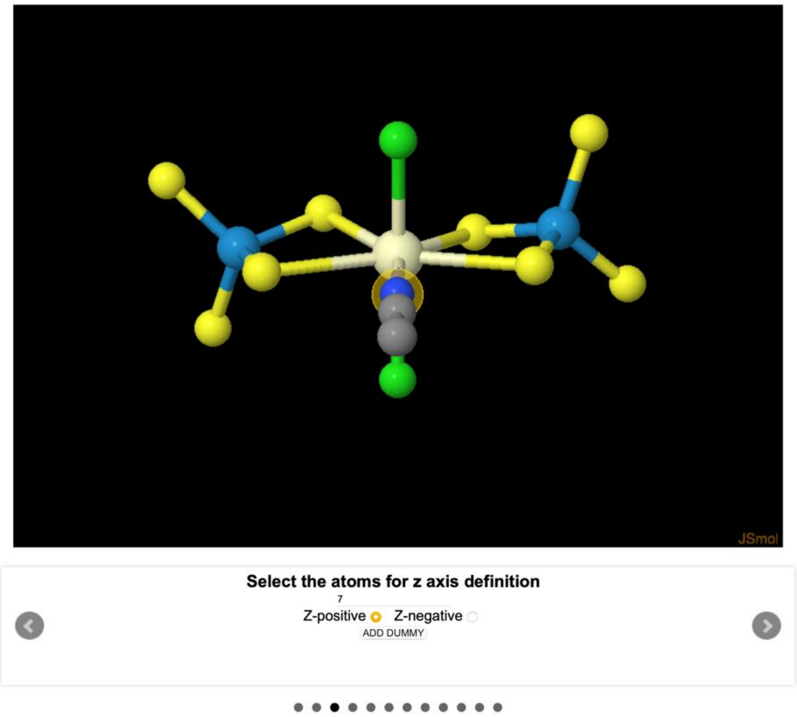

Figure S19: Step 2

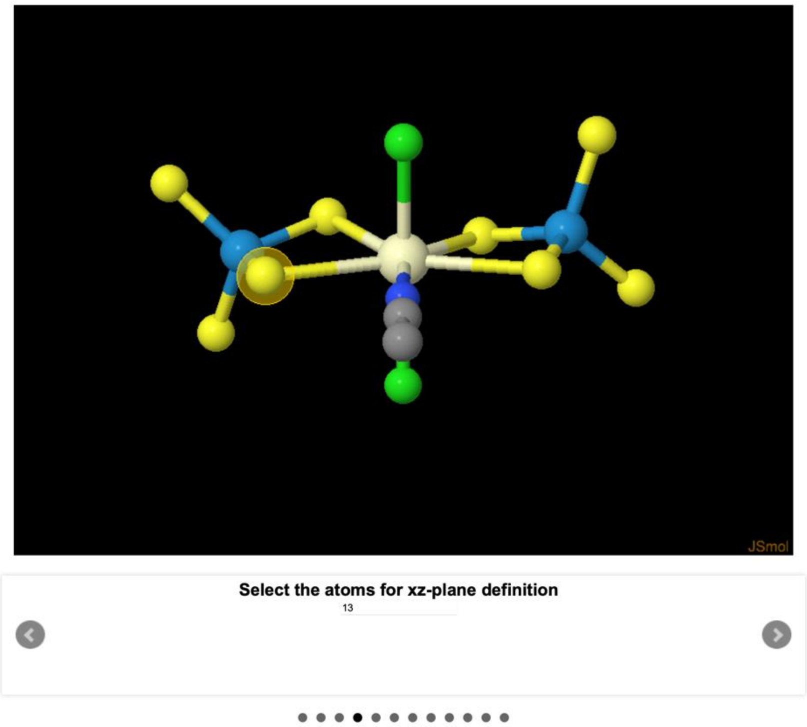

Figure S20: Step 3

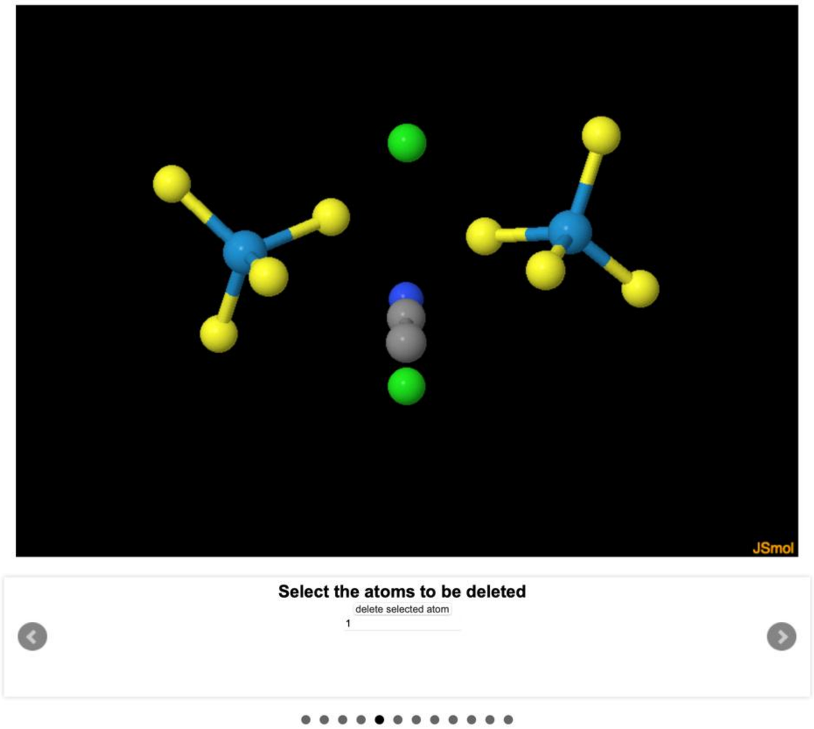

Figure S21: Step 4

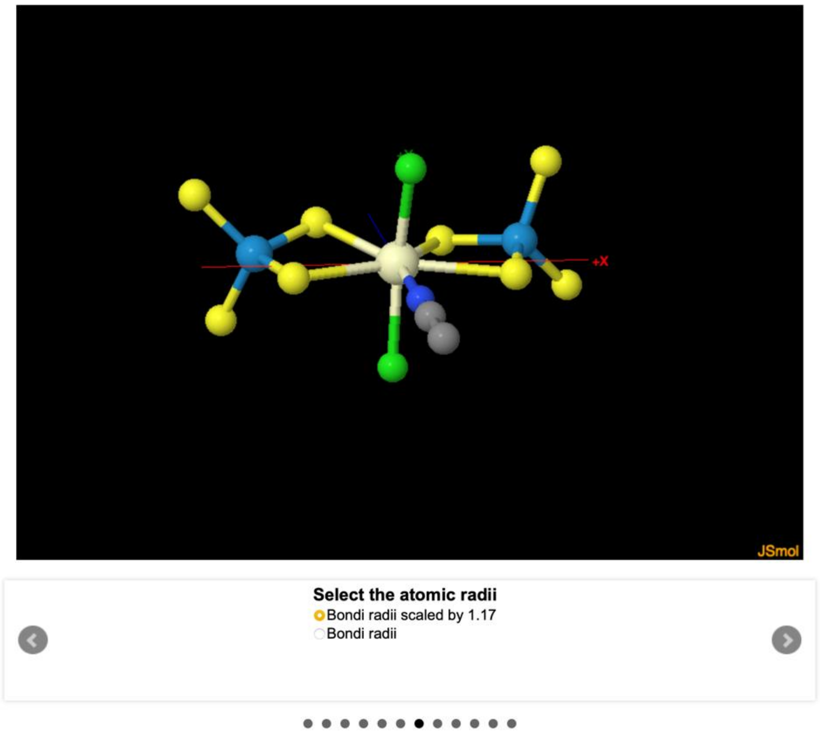

Figure S22: Step 5

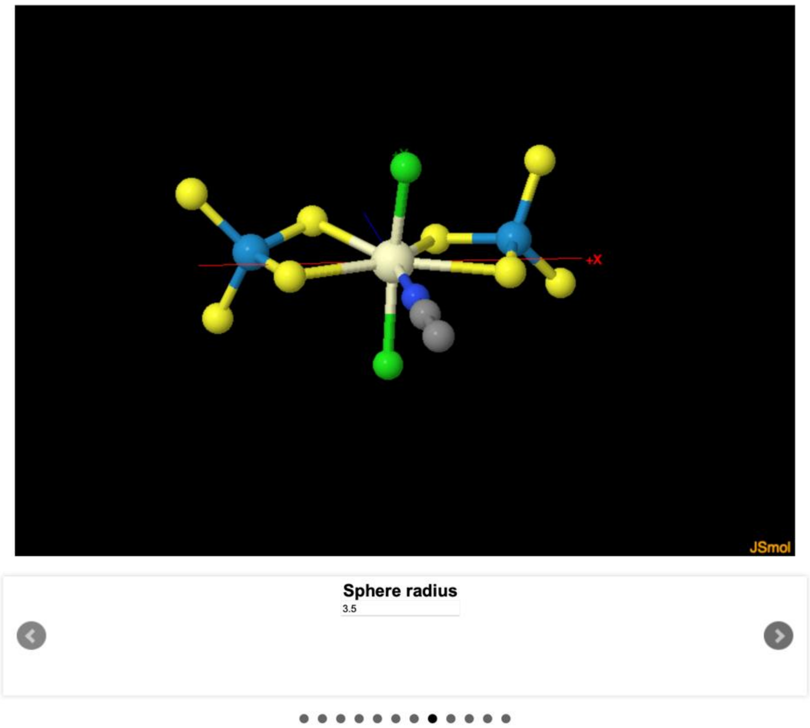

Figure S23: Step 6

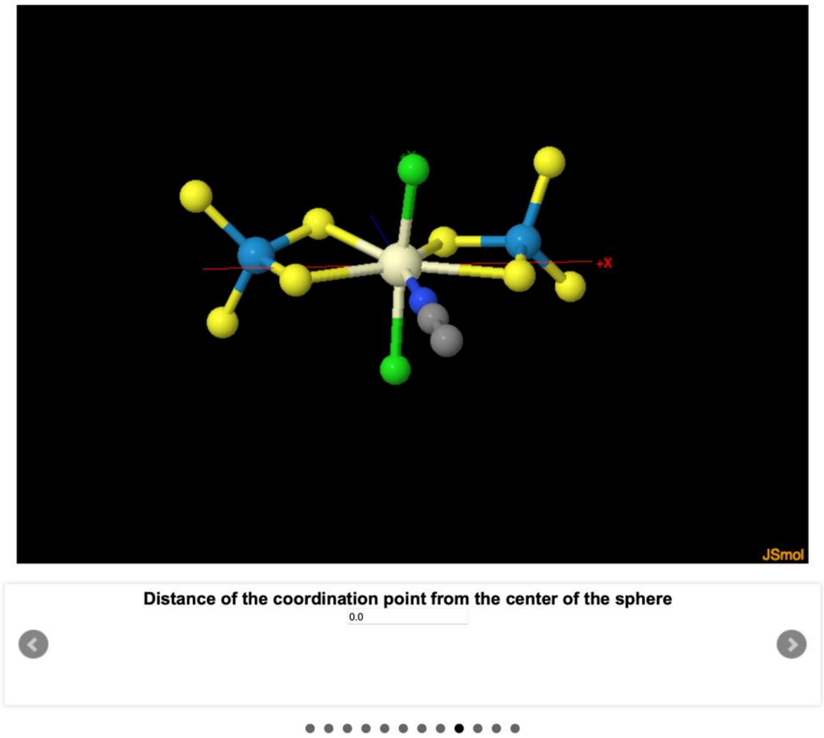

Figure S24: Step 7

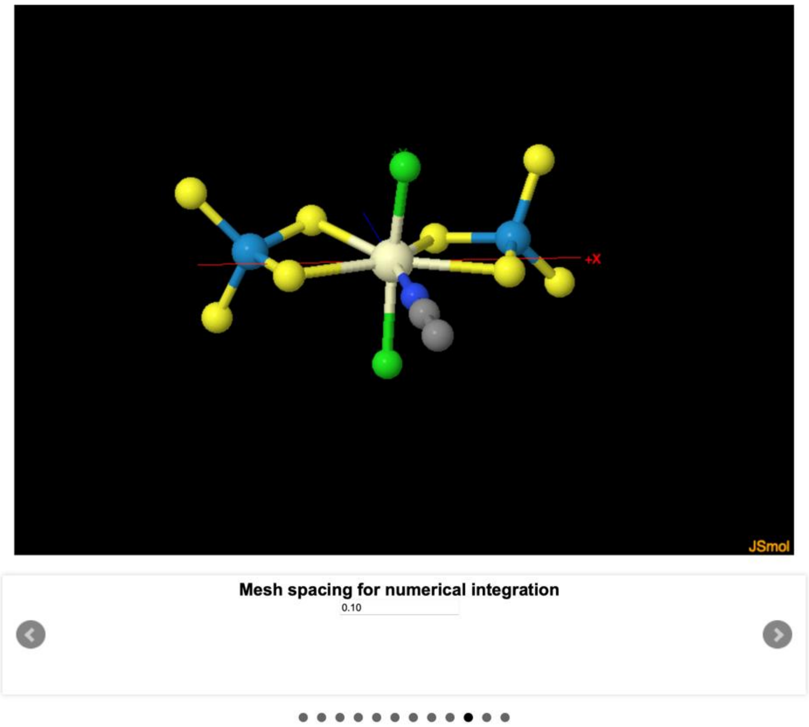

Figure S25: Step 8

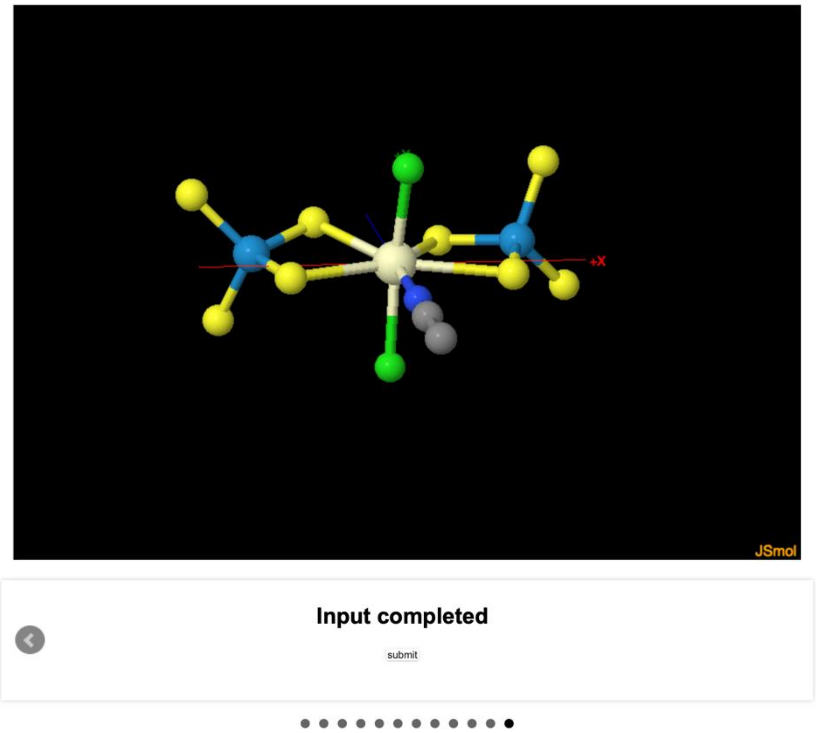

Figure S26: Step 9

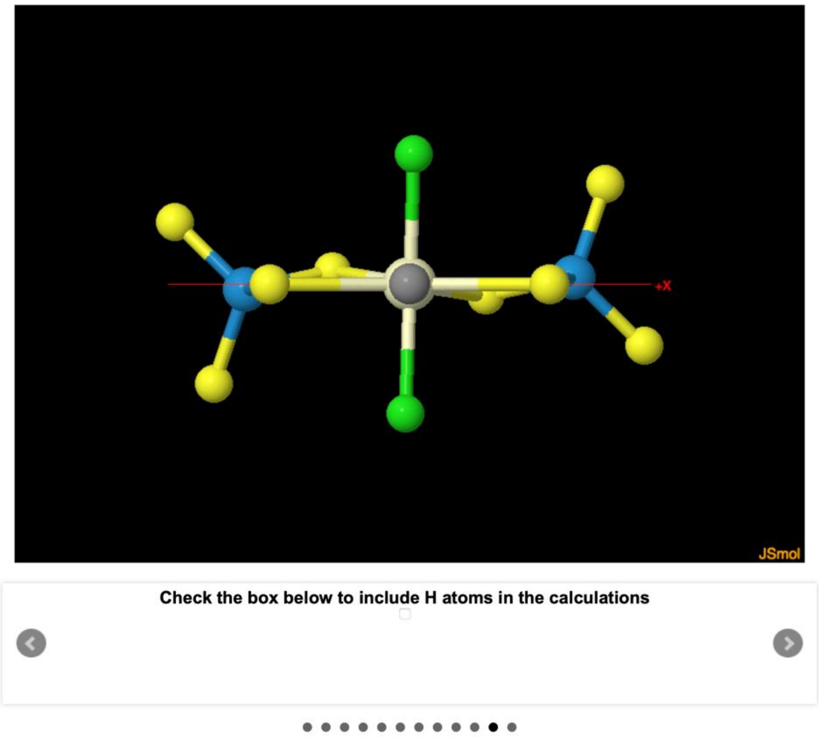

Figure S27: Step 10

For complex **2La** the following procedure was followed:

1. For the center of the sphere, the one lanthanum atom was selected.
2. For the definition of the z axis, the bridging sulfur atom between the two lanthanum atoms was selected.
3. For the definition of the xz-plane, the other lanthanum atom was selected
4. The lanthanide atom that was set as the center was then selected to be deleted.
5. The chosen orientation was checked to ensure that the z axis goes in between the two chloride atoms and that the x axis goes along the Ln-Ln bond
6. The Bondi radii was not scaled.
7. The sphere radius was set to 3.0 Å to calculated $\%V_{bur1}$ and 5.0 Å for $\%V_{bur2}$
8. The distance of the coordination point from the center of the sphere was set to 0.0.
9. The mesh spacing for numerical integration was set to 0.1 Å.
10. H atoms were not included in the calculation.

Table S8: Compared $\%V_{bur}$ in the first ($\%V_{bur1}$, R=3.5 Å) and second coordination sphere ($\%V_{bur1}$, R=5.0 Å) for seven-coordinated $\{DyCl_2\}$ complexes.

N°	Structure	$\%V_{bur1}$ (R=3.0 Å)	$\%V_{bur2}$ (R=5.0 Å)	Delta Buried Volume ($\%V_{bur1}$ - $\%V_{bur2}$)	Steric Map $\%V_{bur2}$ (Cl-Dy-Cl on x axis) (R=3.5 Å)
1[12]	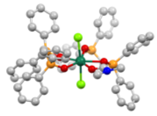	71.4	58.0	13.4	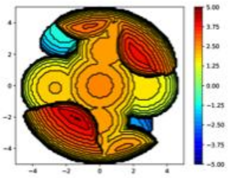
2[13]	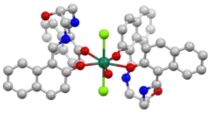	71.7	52.8	18.9	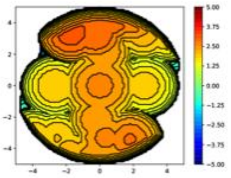
3[14]	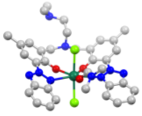	71.8	51.8	20.0	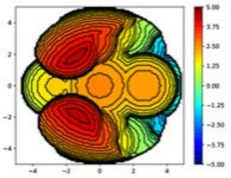
4[15]	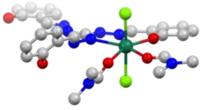	70.3	44.2	26.1	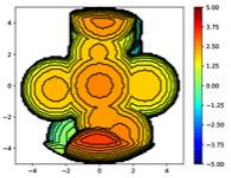
5[16]	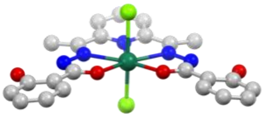	68.9	41.4	27.5	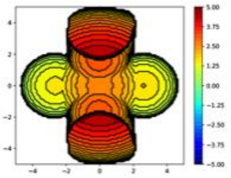
6[17]	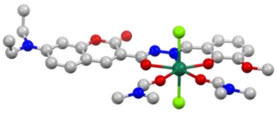	69.8	41.3	28.5	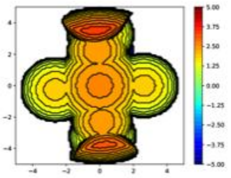
**This work**	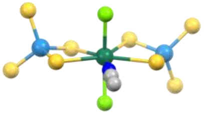	**71.0**	**42.9**	**28.1**	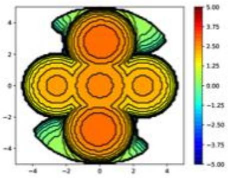

Table S9: Compared structural features and magnetic properties of seven-coordinated $\{DyCl_2\}$ complexes and 1Dy.

N°	Structure	Cl-Dy-Cl	g_z	SMM H = 0	U_{eff} (cm^{-1})	Ref.
1	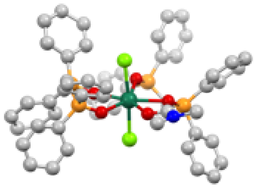	175.12	15.5	NO	-	[12]
2	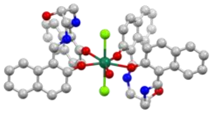	179.13	-	YES	107.7	[13]
3	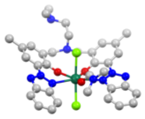	172.55	-	YES	15	[14]
4	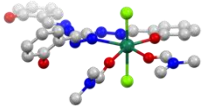	173.7	19.6	NO	-	[15]
5	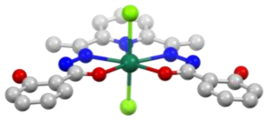	166.33	-	YES	48.6	[16]
6	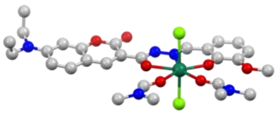	172.05	19.7	YES	31.8	[17]
**1Dy**	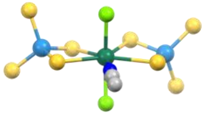	**164.53**	**19.9**	**Yes**	**595**	This work

5. Solid State Luminescence Measurements

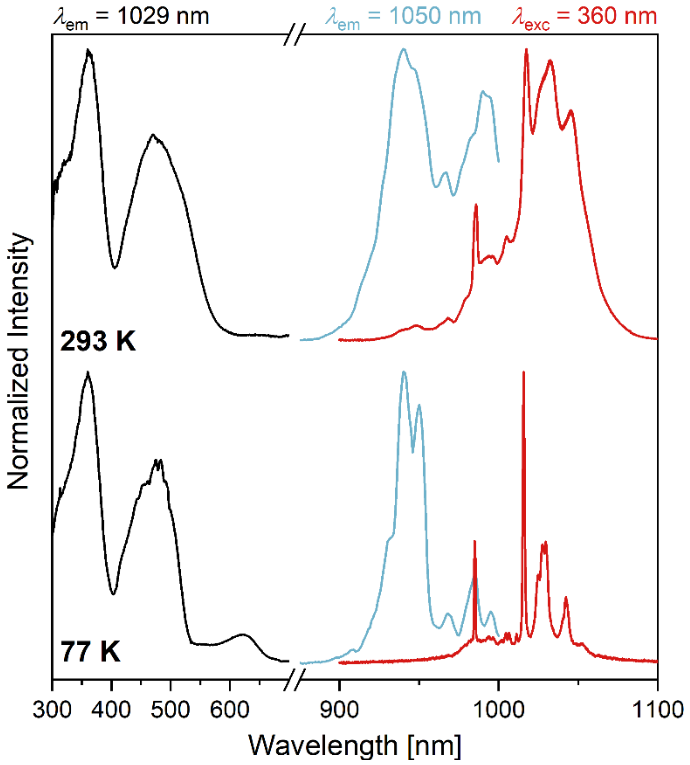

Figure S28: Normalized excitation (λ_{em}=1029 nm, black; λ_{em}=1050 nm blue) and emission (λ_{exc}=360 nm, red) of crystalline **1Yb** samples at 293 K (top) and 77 K (bottom).

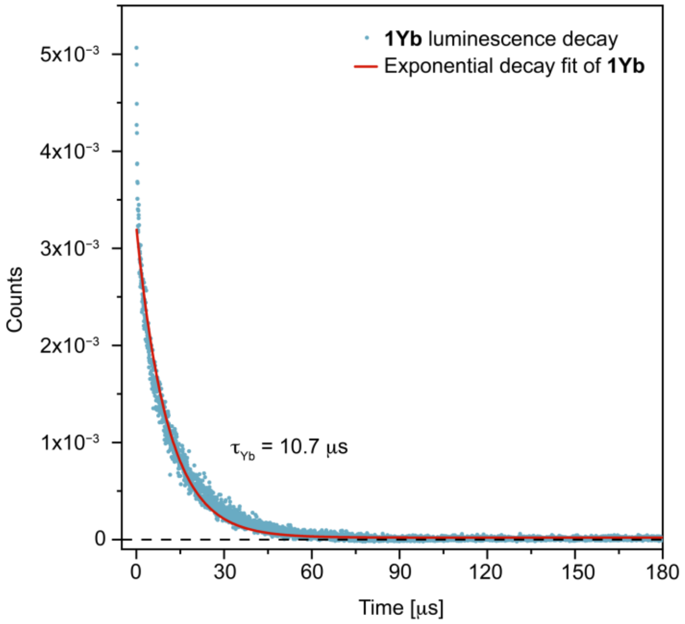

Figure S29: The NIR emission decay (λ_{exc}=360 nm, λ_{em}=1016 nm, blue) and the monoexponential fit (red) of **1Yb** crystals at 293 K. R^2 (COD)=0.9761, reduced χ^2=6.46868·10^{-9}.

6. Magnetic Measurements

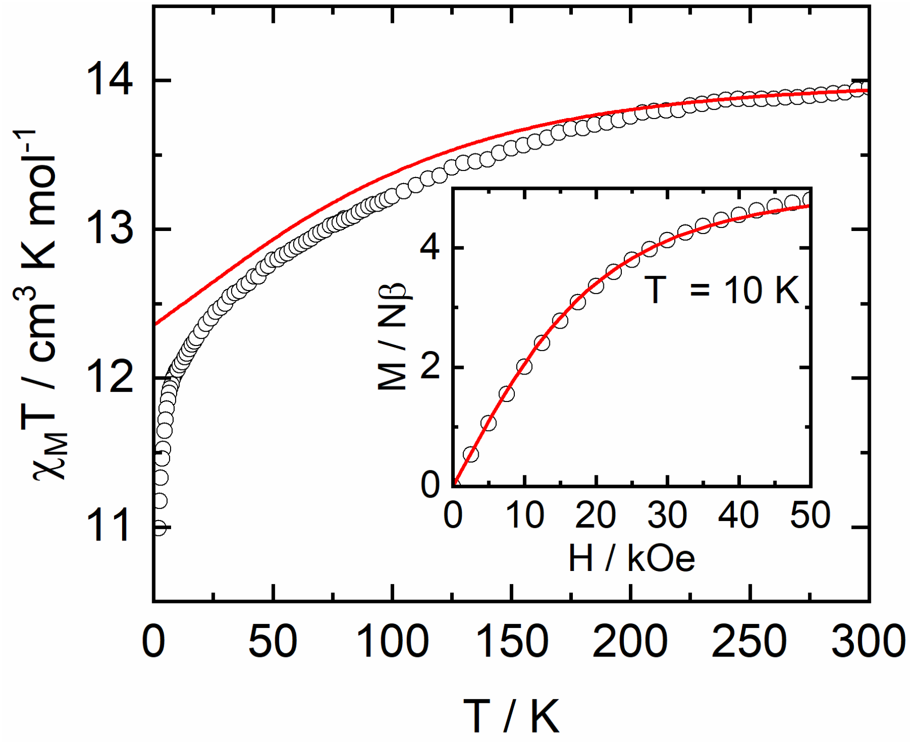

Figure S30: Experimental thermal variation of $\chi_M T$ for **1Dy** (open circles) with calculated curve (see text) in full red line. Inset: field variation of the magnetization of **1Dy** at 2 K (open circles) with calculated curve (see text) in full red line.

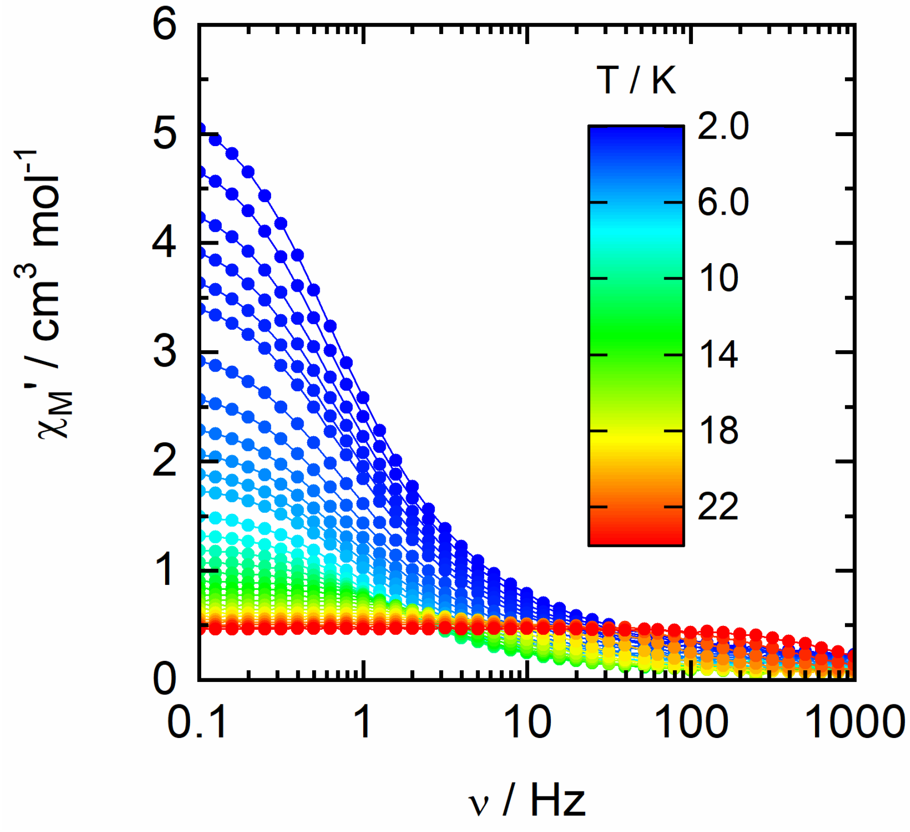

Figure S31: Experimental variation of the in-phase component (χ_M') of the ac susceptibility as a function of the frequency of the oscillating alternating magnetic (3 Oe amplitude) measured in zero external constant field for **1Dy** at various temperatures between 2 and 24 K.

Extended Debye model.

$$\chi' = \chi_S + (\chi_T - \chi_S)\frac{1+(\omega\tau)^{1-\alpha}\sin\left(\alpha\frac{\pi}{2}\right)}{1+2(\omega\tau)^{1-\alpha}\sin\left(\alpha\frac{\pi}{2}\right)+(\omega\tau)^{2-2\alpha}}$$

$$\chi'' = (\chi_T - \chi_S)\frac{(\omega\tau)^{1-\alpha}\cos\left(\alpha\frac{\pi}{2}\right)}{1+2(\omega\tau)^{1-\alpha}\sin\left(\alpha\frac{\pi}{2}\right)+(\omega\tau)^{2-2\alpha}}$$

With χ_T the isothermal susceptibility, χ_S the adiabatic susceptibility, τ the relaxation time and α an empiric parameter which describe the distribution of the relaxation time. For SMM with only one relaxing object α is close to zero. The extended Debye model was applied to fit simultaneously the experimental variations of χ_M' and χ_M'' with the frequency ν of the oscillating field ($\omega = 2\pi\nu$). Typically, only the temperatures for which a maximum on the χ_M'' vs. ν curves, have been considered (see figure here below for an example). The best fitted parameters τ, α, χ_T, χ_S are listed in Tables here below with the coefficient of determination R^2.

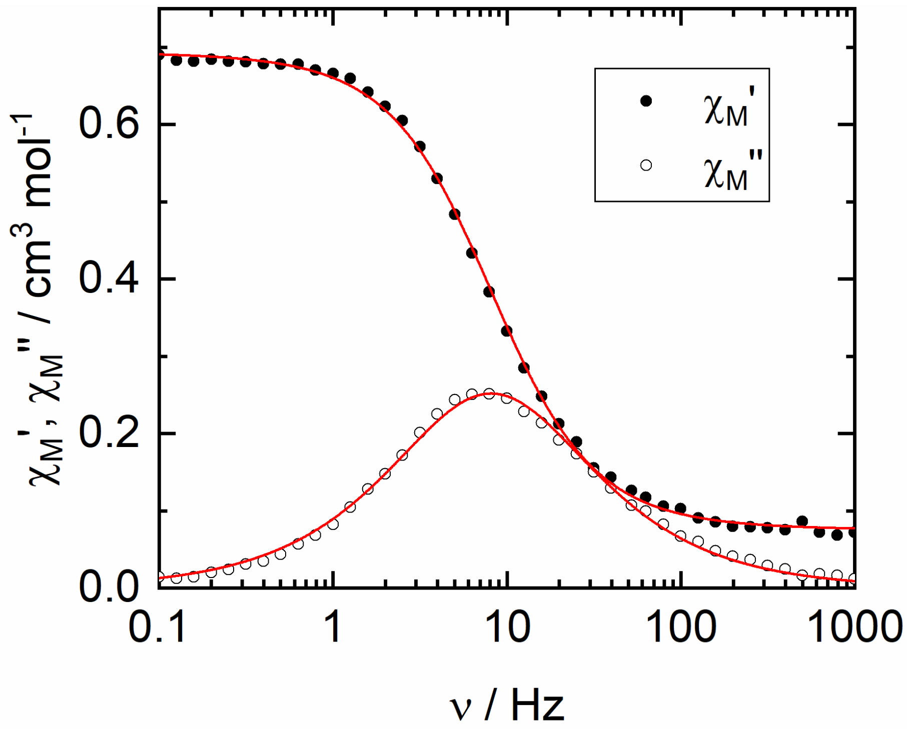

Figure S32: Frequency dependence of the in-phase (●) and out-of-phase (○) components of the ac susceptibility measured on powders at 16 K in zero dc field with the best fitted curves (red lines) for compound **1Dy**. Best fitted parameters are reported in Tables here below.

Table S10. Best fitted parameters (χ_T, χ_S, τ and α) with the extended Debye model for **1Dy** at 0 Oe in the temperature range 2-24 K. Only temperatures for which a maximum is visible on χ_M'' vs. ν curves have been considered in order to not overparametrize fitting procedures.

T / K	χ_T / cm^3 mol^{-1}	χ_S / cm^3 mol^{-1}	α	τ / s	R^2
2	5.49769	0.31219	0.25407	0.1892	0.99845
2.2	5.05538	0.29724	0.25482	0.1846	0.99847
2.4	4.59163	0.29105	0.25215	0.18056	0.99861
2.6	4.2352	0.26752	0.25423	0.17608	0.99856
2.8	3.92707	0.26289	0.25208	0.17332	0.99862
3	3.66639	0.24954	0.25221	0.17051	0.99865
3.5	3.14548	0.22326	0.25199	0.16419	0.99864
4	2.75455	0.20655	0.25043	0.15981	0.99864
4.5	2.44768	0.18783	0.24917	0.15526	0.9987
5	2.20917	0.17261	0.24907	0.15225	0.99878
5.5	2.01189	0.16152	0.24819	0.14821	0.99877
6	1.84433	0.14889	0.24789	0.14317	0.99854
7	1.5843	0.13606	0.24143	0.13381	0.99875
8	1.38457	0.12323	0.22578	0.11987	0.99865
9	1.25386	0.11956	0.22131	0.10858	0.99745
10	1.1292	0.10556	0.21293	0.08853	0.99861
11	1.02044	0.09943	0.19184	0.07025	0.99884
12	0.9271	0.0928	0.17382	0.05365	0.99897
13	0.85447	0.08565	0.15981	0.04161	0.99915
14	0.7928	0.08104	0.1492	0.03233	0.99905
15	0.74017	0.07684	0.13871	0.02525	0.99899
16	0.69488	0.07116	0.13648	0.0194	0.99832
17	0.65516	0.06858	0.13292	0.01465	0.99895
18	0.61724	0.07256	0.11513	0.01074	0.99935
19	0.58728	0.0647	0.12385	0.00691	1
20	0.55622	0.06849	0.10433	0.00396	0.99946
21	0.53082	0.07727	0.10073	0.0021	0.99863
22	0.50897	0.06717	0.11754	9.8458E-4	0.99946
23	0.48629	0.07152	0.10304	4.68339E-4	0.99953
24	0.46784	0.07101	0.08751	2.3147E-4	0.99668

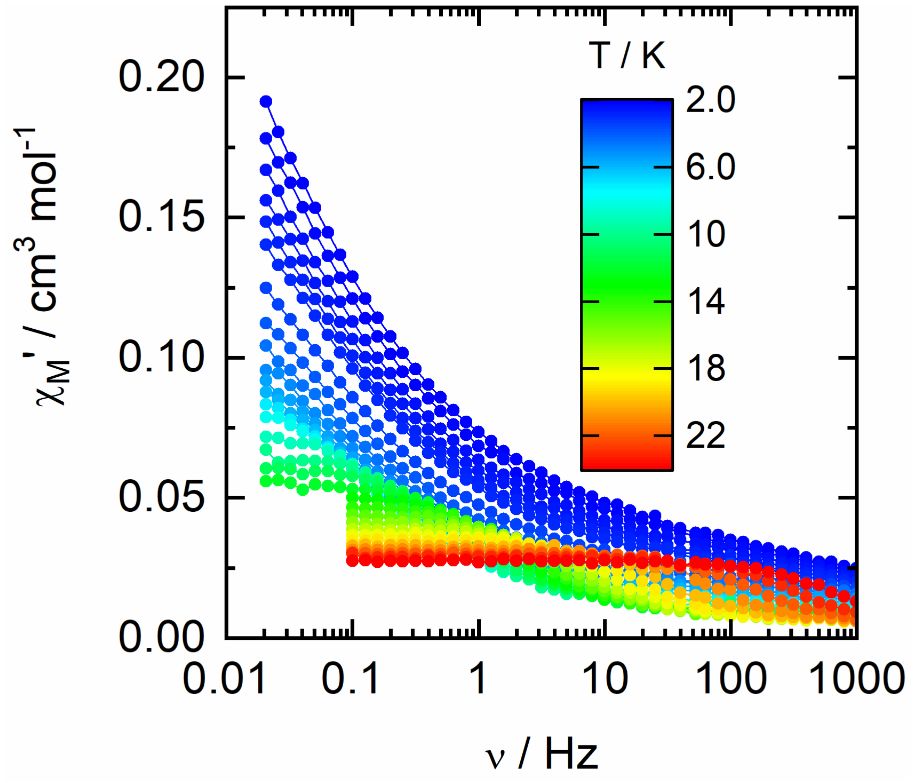

Figure S33: Experimental variation of the in-phase component (χ_M') of the ac susceptibility as a function of the frequency of the oscillating alternating magnetic (3 Oe amplitude) measured in zero external constant field for **1Dy@Y** at various temperatures between 2 and 24 K.

Table S11. Best fitted parameters (χ_T, χ_S, τ and α) with the extended Debye model for **1Dy@Y** at 0 Oe in the temperature range 2-24 K. Only temperatures for which a maximum is visible on χ_M'' vs. ν curves have been considered in order to not overparametrize fitting procedures.

T / K	χ_T / cm^3 mol^{-1}	χ_S / cm^3 mol^{-1}	α	τ / s	R^2
2	0.34339	0.03678	0.54247	8.15489	0.99962
2.2	0.31078	0.03532	0.5308	6.91469	0.99958
2.4	0.28048	0.03315	0.52027	6.06218	0.99961
2.6	0.25602	0.03141	0.51218	5.37177	0.99968
2.8	0.23523	0.02982	0.50249	4.75148	0.99967
3	0.22021	0.02799	0.5005	4.46086	0.99974
3.5	0.18662	0.02493	0.48675	3.6367	0.99973
4	0.16362	0.02231	0.47824	3.13943	0.99974
4.5	0.14778	0.02061	0.4704	2.89891	0.99967
5	0.13698	0.01904	0.46421	2.77635	0.99939
5.5	0.132	0.01465	0.49798	2.74044	0.99847
6	0.12417	0.01368	0.48716	2.46865	0.99861
7	0.10542	0.01289	0.42844	1.51646	0.99815
8	0.09047	0.0119	0.36918	0.87388	0.99757
9	0.07689	0.01124	0.29696	0.47405	0.99729

10	0.06745	0.01058	0.24404	0.28438	0.99754
11	0.0618	0.00966	0.22767	0.18144	0.99881
12	0.05582	0.00875	0.20058	0.11307	0.99817
13	0.05163	0.00838	0.19053	0.07884	0.9986
14	0.04767	0.0078	0.17411	0.05344	0.99861
15	0.04422	0.00738	0.15725	0.03798	0.99884
16	0.04169	0.00673	0.16912	0.02707	0.99903
17	0.03918	0.00655	0.15257	0.02001	0.99901
18	0.03698	0.00623	0.15611	0.01352	0.99881
19	0.03501	0.00619	0.14283	0.00842	0.99903
20	0.033	0.00597	0.13117	0.00471	0.99843
21	0.03144	0.0051	0.16963	0.00218	0.99855
22	0.03008	0.00547	0.14551	0.00111	0.99882
23	0.02886	0.00278	0.18984	4.35643E-4	0.99869
24	0.02758	0.00207	0.20082	2.00296E-4	0.9989

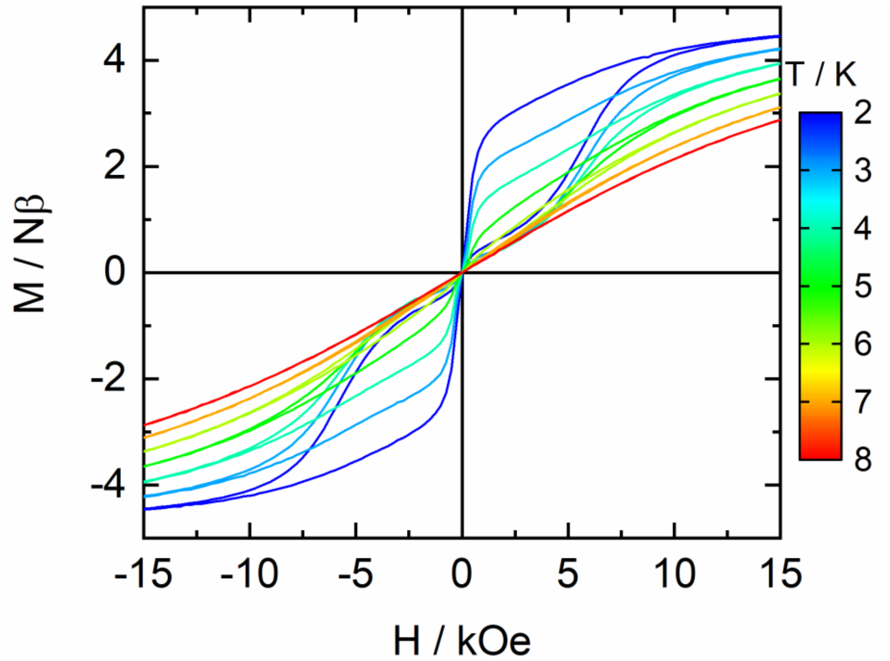

Figure S34: Hysteresis loops for **1Dy** measured at a sweep rate of 16 Oe s^{-1} at various temperatures.

7. Computational Details

Wavefunction-based calculations were carried out on molecular structures of the **1Dy**, and **1Yb** complexes by using the SA-CASSCF/RASSI-SO approach, as implemented in the OpenMolcas quantum chemistry package (version 22.10).[18-19] In this approach, the relativistic effects are treated in two steps on the basis of the Douglas–Kroll Hamiltonian.[20-22] First, the scalar terms were included in the basis-set generation and were used to determine the spin-free wavefunctions and energies in the complete active space self-consistent field (CASSCF) method.[23] For **1Dy**, the active space consisted of the nine 4f electrons of the Dy(III) ion spanning the seven 4f orbitals, i.e. CAS(9,7)SCF while for **1Yb**, the active space consisted of the thirteen 4f electrons of the Yb(III) ion spanning the seven 4f orbitals, i.e. CAS(13,7)SCF. In order to obtain a better description of the electronic structure of **1Yb**, calculations were also computed using perturbation theory at the second order, i.e. CAS(13,7)PT2, based on the previous state average CAS(13,7)SCF descriptions.[24] Next, spin-orbit coupling was added within the restricted-active-space-state-interaction (RASSI-SO) method, which uses the spin-free wavefunctions as basis states.[25-26] Spin−orbit (SO) integrals are calculated using the AMFI (atomic mean- field integrals) approximation.[27] The resulting wavefunctions and energies are used to compute the magnetic properties and g-tensors of the lowest states from the energy spectrum by using the pseudo-spin S = 1/2 formalism in the SINGLE-ANISO routine.[28-29] Decomposition of the bielectronic integrals was employed to save disk space and speed-up the calculations.[30] For **1Dy**, state-averaged CASSCF calculations were performed for all of the sextets (21 roots), all of the quadruplets (224 roots), and 300 out of the 490 doublets of the Dy^{III} ion. 21 sextets, 128 quadruplets, and 107 doublets were mixed through spin−orbit coupling in RASSI-SO. For **1Yb**, state-averaged CASSCF calculations were performed for the 7 doublets. All atoms were described by ANO-RCC basis sets.[31-33] The following contractions were used: [8s7p5d3f2g1h] for Dy and Yb, [7s6p4d2f] for W, [5s4p2d] for S and Cl, [4s3p2d] for N and C, and [2s] for H. The atomic positions were extracted from the X-ray crystal structures. Only the positions of the H atoms were optimized with the AMS program suite (revision 2022.103).[34-35] These calculations utilized the scalar all-electron zeroth-order regular approximation (ZORA)[36-37] along with the revPBE (Perdew-Burke-Ernzerhof) functional.[38-39] For all atoms, the atomic basis set corresponded to the triple-ζ polarized (TZP) Slater-type orbital (STO) all-electron basis set.[40]

To give more insights into the orientation of the magnetic axis, the molecular electrostatic potential is calculated from the LOPROP charge analysis[41]:

$$V(\vec{r_i}) = \sum_{i}^{N} \frac{\vec{q_i}}{|\vec{r_i} - \vec{r}|} + \frac{\vec{p_i} \cdot \vec{r_i}}{|\vec{r_i} - \vec{r}|^3} + \frac{\vec{r_i} \cdot (\vec{Q_i} \times \vec{r_i})}{|\vec{r_i} - \vec{r}|^5}$$

Where q_i, p_i, Q_i and r_i are respectively the charge, dipole, quadrupole moments, and displacement vector of the i -th atom. The resulting molecular electrostatic potential is mapped

using the home-made CAMMEL code (Calculated Molecular Multipolar Electrostatics).[42-44] The CAMMEL code is available under GNU General Public License v3.0 and can be downloaded at https://github.com/rmarchal1/CAMMEL.

Table S12: Relative energies (cm^{-1}) for the ground and excited Kramers doublets (KD) of **1Yb** obtained at both SA-CAS(13,7)SCF/RASSI-SO and SA-CAS(13,7)PT2/RASSI-SO levels.

	KD	SA-CAS(13,7)SCF	SA-CAS(13,7)PT2
$^2F_{5/2}$	2’	10634	10753
	1’	10548	10588
	0’	10322	10343
$^2F_{7/2}$	3	378	507
	2	298	361
	1	205	243
	0	0	0

Table S13: Calculated energy levels of the four Kramers Doublets (KD) of the ground state multiplet $^2F_{7/2}$ of complex **1Yb** with g-tensor eigenvalues and wavefunction composition on the basis of M_J states of the ground state multiplet. Only contributions higher than 10% to wavefunction are explicitly mentioned.

KD	Energy (cm^{-1})	g			Wavefunction
SA-CAS(13,7)SCF/RASSI-SO					
0	0	0.2	0.4	7.7	96.6|±7/2>
1	205	1.9	3.1	4.8	60.9|±5/2>+33.4|±3/2>
2	298	0.0	1.5	5.8	36.2|±3/2>+32.1|±1/2>+31.1|±5/2>
3	378	0.1	1.0	7.0	60.3|±1/2>+29.8|±3/2>
SA-CAS(13,7)PT2/RASSI-SO					
0	0	0.1	0.8	7.3	91.8|±7/2>
1	243	0.2	2.0	5.9	56.7|±3/2>+27.7|±5/2>+14.9|±1/2>
2	361	4.0	2.4	0.1	50.9|±1/2>+41.2|±5/2>
3	507	0.3	1.9	5.7	39.7|±3/2>+30.9|±5/2>+26.8|±1/2>

Table S14: Calculated energy levels of the eight Kramers Doublets (KD) of the ground state multiplet $^6H_{15/2}$ of complex **1Dy** with g-tensor eigenvalues and wavefunction composition on the basis of M_J states of the ground state multiplet. Only contributions higher than 10% to wavefunction are explicitly mentioned.

KD	Energy (cm^{-1})	g			Wavefunction
1	0.0	0.0	0.0	19.9	99.9|±15/2>
2	203	0.0	0.0	17.0	98.3|±13/2>
3	316.8	9.7	7.0	2.1	46.1|±11/2>+19.7|±3/2>+16.1|±1/2>+13.4 |±7/2>
4	329.8	6.9	6.1	1.6	39.6|±1/2>+28.8|±11/2>+14.4|±5/2>+10.0 |±3/2>
5	364.3	9.9	6.2	1.3	27.9|±3/2>+24.0|± 9/2>+23.4|± 5/2>+14.1|±11/2>
6	405.5	1.4	2.6	14.1	31.7|±9/2>+26.1|±1/2>+15.7|±3/2>+13.2|±7/2>
7	419.9	0.4	0.4	19.2	30.0|±7/2>+25.1|±5/2>+18.9|±9/2>+15.2|±3/2>
8	469.2	0.0	0.0	19.0	36.8|±7/2>+27.5|±5/2>+18.8|±9/2>+11.4|±3/2>

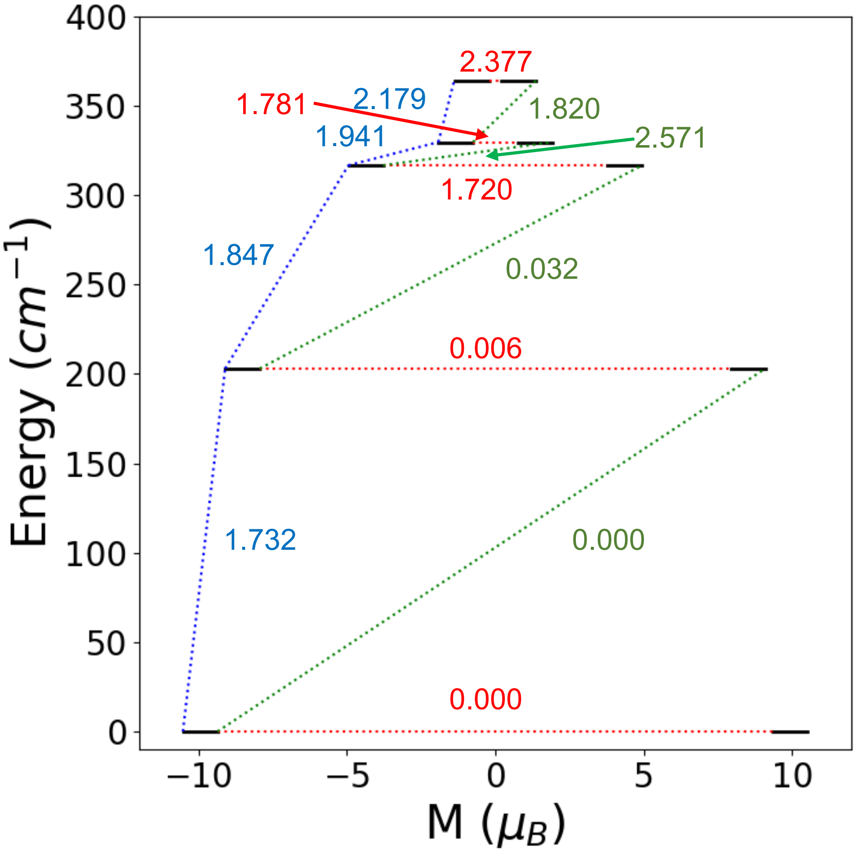

Figure S35: Calculated energies of the five first KD of the complex **1Dy**. Colored dashed lines with numbers correspond to the transition moments between electronic states, directly related to the probability the event occurs.

Additional Quantum mechanical calculations were carried out using the ORCA v. 6.0.1 program package.[45-47] Specifically, the DFT-optimized geometric parameters of the tris anion **3La** and of the hypothetical monomeric La chloride complex, **1La**, were explored. To this end, the *revPBE* functional,[48] in conjunction with the *ZORA-def2-TZVP* (for Br/Cl, S, N, C, H) and *SARC-ZORA-TZVP* (for La & W) basis sets were used.[49] Also, Grimme's D3 correction complemented by the Becke-Johnson damping function was used to account for dispersion (*D3BJ*),[50-51] and the Resolution-of-Identity approximation (*RI*) with the appropriate auxiliary basis set (*SARC/J*) was implemented in order to speed up the calculations.[52] Tighter-than-default grid settings (*DEFGRID3*), as well as tighter-than-default convergence criteria were enforced for the geometric (*tightopt*) and SCF iterations (*tightscf*). All structure optimizations were accompanied by an analytical frequency calculation (*AnFreq*) to establish the final geometry as a true minimum on the potential energy surface by absence of imaginary frequencies.

4 initial geometries were tested for the 2 complexes in question:

(1) Optimization of **3La** from the single crystal XRD coordinates of **3La**;
(2) Optimization of **3La** using the single crystal XRD coordinates of **1Ce**, substituting Cl for Br;
(3) Optimization of **1La** using the single crystal XRD coordinates of **3La**;
(4) Optimization of **1La** using the single crystal XRD coordinates of **1Ce**, substituting Br for Cl;

In this 2x2 set, both respective starting points (1/3 or 2/4) yielded the very same endpoints of the two optimized geometries for **3La** and **1La**. In line with the observed extensive intermolecular interactions in the crystal lattices of the **1Ln**-series, the final geometries evidenced detachment of the MeCN ligand from the monomeric halide complexes (Figure S36a,b)—a fair indication for the fact that these very weak intermolecular C-H···S and C-H···Cl interactions in the crystal packing scheme are essential for the structural integrity of the complexes.

Conversely, we then set out to test the effect of various constraints on these geometry optimization runs: Specifically, keeping the Ln-N-C angle, the Ln-N bond distance or both fixed at their respective experimental (single crystal XRD-derived) values. It was thereby found that constraining the Ln-N-C angle was enough of a constraint to maintain the MeCN ligand bound to the Ln atom, yielding the optimized structures shown in Figures S36c,d.

Notably, for **3La**, the corresponding DFT-optimized geometry is very similar to that determined via single crystal XRD, with deviations in the individual bond-lengths that are within typical error of the DFT-approach.

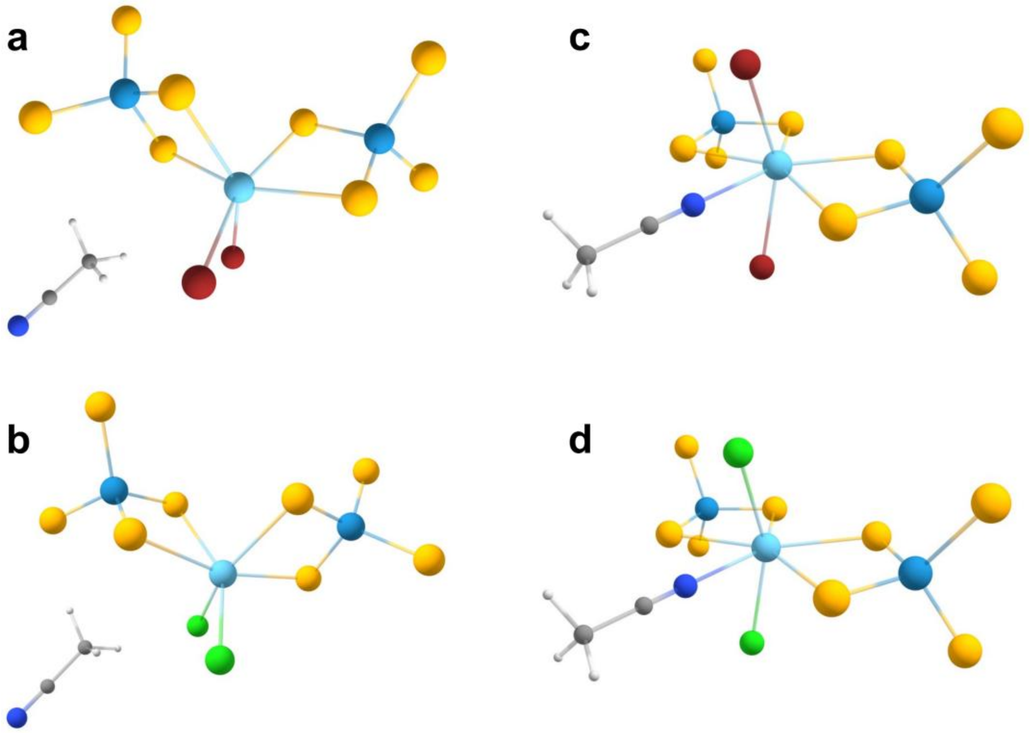

Figure S36: DFT-optimized geometries of **3La** (panels a,c) and **1La** (panels b,d), with (c,d) and without (a,b) constraining the respective Ln-N-C angles.

Comparing the single-point energies of the 4 structures with one another, it can be concluded that DFT predicts a significant energetic stabilization upon de-coordination of the MeCN ligand from either complex (113 kJ mol^{-1} in **3La** vs. 117 kJ mol^{-1} in **1La**). The difference between these two energies for MeCN-de-coordination is small (ca. 4 kJ mol^{-1}), and predicts MeCN-detachment to occur favorably in chloride-bound **1La**, with respect to bromide-bound **3La**.

This being said, from a more practical viewpoint, it nonetheless appears plausible that crystal packing effects and the large excess of MeCN molecules present in dissolved samples of the complexes should lead to the exclusive observation of MeCN-bound species in all the experiments described throughout this work.

Table S15: Buried Volume calculated at a radius of 3 and 5 Å for optimized geometries 1-4 (see section 6 for detailed on the calculation).

Optimized Geometry	1	2	3	4
$\%V_{bur1}$ (R=3 Å)	61.1	61.1	60.7	60.7
$\%V_{bur2}$ (R=5 Å)	42.4	42.4	40.6	40.6
Delta $\%V_{bur}$	18.7	18.7	20.1	20.1

The optimized geometric parameters for all 4 structures are condensed below :

Figure S36a :

W	12.69008731204566	10.16050357230660	26.18065884256276
La	9.16266623869526	11.41735446484176	25.91297403225038
Br	8.14443735805197	9.76253166858960	23.68791158684698
S	10.79548860470856	9.08004954295407	26.66411923535487
S	12.02831016776550	12.23808033273230	25.70911629154476
S	14.07184382752779	10.13492541272299	27.88292454303510
S	13.65396765682876	9.27514596925194	24.41208456743448
N	12.05870960375258	11.63793906210578	19.66984507478229
C	11.72966540978105	11.51232681939091	20.78478352567109
W	7.03741804862451	11.95135602393732	28.95146188268723
Br	8.90982446699767	13.86063922201580	24.31516714041569
S	9.12571864008811	12.65921632965109	28.58538676968968
S	6.53013249668105	10.78849751662483	27.10754523440452
S	6.95226199303130	10.66319067269240	30.72519891766770
S	5.66813773239860	13.64992542938641	29.19344852019513
C	11.32433500844954	11.35644515401666	22.17225267959477
H	11.09606345855780	12.33315291145285	22.62398571545830
H	10.41520178674295	10.73787098847114	22.24811483307747
H	12.12073018926975	10.87047490685382	22.76119260732303

Figure S36b :

W	2.83330602525523	17.61604409840354	6.69273429044502
La	4.54647826284240	20.64346744355084	8.14358432424664
S	5.01912743987843	17.92195655001388	7.03426783239125
Cl	5.35983948547551	22.13619929592933	5.99294330419348

S	1.88685861902823	19.48650428909967	7.47697136052196
S	2.40408385884561	17.37215945435112	4.55197043452397
S	2.11067859994365	15.86454225944074	7.80119118184548
N	7.00481373234847	26.89855306181266	9.30071316944520
C	6.72958460312450	25.76270553057757	9.26233035935604
W	7.17012586349480	20.37170602280749	10.85153664500533
S	5.00369843868433	19.85429011766771	10.98229203991860
Cl	3.03943031378933	22.73039306110209	9.15074527916856
S	7.49374893426065	20.85525065137821	8.69832987388983
S	7.61597747948612	22.14343093236114	12.08148392747464
S	8.42605948111486	18.68604759690821	11.48040404786255
C	6.39457869855793	24.34871202831534	9.21824486545993
H	5.30389377204549	24.20028153782371	9.27517941835209
H	6.73985664114975	23.89596546735097	8.27763455251917
H	6.86294975067382	23.80757660110238	10.05874509337890

Figure S36c :

W	13.78937674392107	10.63889400240393	27.34742008063455
La	10.18112375345845	11.26893443210066	26.29485988468405
Br	9.69292128711093	8.45941370487159	25.49133306793090
S	11.97716444913850	10.94487071335848	28.63112333667038
S	13.03177355655533	10.78090711457044	25.26088690646221
S	15.28996221979296	12.19889372045309	27.74093815040686
S	14.67960019812004	8.66217866625604	27.72178269307085
N	10.18529114173385	11.26808842547109	23.59716583644513
C	10.18708064387219	11.26772530087175	22.43877406647882
W	6.57678008000394	11.89795558061024	27.35818149071250
Br	10.66463840857458	14.07994917658015	25.49582077971195
S	8.39191014553490	11.58896116565403	28.63704276614115
S	7.32964395784574	11.75850797666849	25.26972917264106
S	5.07619767582861	10.33821907287718	27.75278383295331
S	5.68862197295434	13.87466651655905	27.73753233340527
C	10.19177689123572	11.26579904596659	20.98519086082607
H	11.22289428591388	11.16780186535627	20.61421302590745
H	9.59466839575441	10.42217407310209	20.60761537277247
H	9.76357419265034	12.20568544626861	20.60577834214446

Figure S36d :

W	2.47286859122317	19.18318312890170	6.46565873462209
La	5.36290772484028	20.23390588760011	8.75299052231045
S	3.91217707298415	17.89628822523321	7.60392656296177
Cl	7.01681801331230	20.99070261392224	6.66254744032130
S	3.07305842140588	21.27083051151038	6.93862396910906
S	2.63274214108576	18.79738932905152	4.30363734892053
S	0.40578395421673	18.79893545830203	7.12003715839043
N	5.36524443972092	22.95268046254015	8.75450687491895
C	5.36624009793349	24.11119433444509	8.75515308780412
W	8.25553014804525	19.19112634482102	11.04166598654244
S	6.82046061476168	17.90064240527389	9.90208574771603
Cl	3.70592451288914	20.98295908406034	10.84246520822914
S	7.65074411439839	21.27737437123011	10.56843252859439
S	8.09492722820425	18.80455313319450	13.20347333825649
S	10.32408134396866	18.81205813037036	10.38889388311808
C	5.37040870936384	25.56551845109312	8.75632689777919
H	4.34278204794953	25.94542234948062	8.85715602393195
H	5.97298451431498	25.93856145624991	9.59789552288243
H	5.79940630938148	25.94246032271909	7.81582516359111

References

[1] G. M. Sheldrick, « A short history of SHELX » *Acta Crystallogr. A* **2008**, *64*, 112.

[2] G. M. Sheldrick, « Crystal structure refinement with SHELXL » *Acta Crystallogr. C* **2015**, *71*, 3.

[3] G. M. Sheldrick, « SHELXT - integrated space-group and crystal-structure determination » *Acta Crystallogr. A* **2015**, *71*, 3.

[4] M. Llunell, D. Casanova, J. Cirera, P. Alemany, S. Alvarez, « SHAPE: Program for the stereochemical analysis of molecular fragments by means of continuous shape measures and associated tools, University of Barcelona, Barcelona, 2013. » *SHAPE: Program for the stereochemical analysis of molecular fragments by means of continuous shape measures and associated tools, University of Barcelona, Barcelona, 2013.* **2013**.

[5] D. Casanova, J. Cirera, M. Llunell, P. Alemany, D. Avnir, S. Alvarez, « Minimal Distortion Pathways in Polyhedral Rearrangements » *J. Am. Chem. Soc.* **2004**, *126*, 1755.

[6] J. Cirera, E. Ruiz, S. Alvarez, « Shape and Spin State in Four-Coordinate Transition-Metal Complexes: The Case of the d6 Configuration » *Chem. Eur. J.* **2006**, *12*, 3162.

[7] L. Falivene, R. Credendino, A. Poater, A. Petta, L. Serra, R. Oliva, V. Scarano, L. Cavallo, « SambVca 2. A Web Tool for Analyzing Catalytic Pockets with Topographic Steric Maps » *Organometallics* **2016**, *35*, 2286.

[8] L. Falivene, Z. Cao, A. Petta, L. Serra, A. Poater, R. Oliva, V. Scarano, L. Cavallo, « Towards the online computer-aided design of catalytic pockets » *Nat. Chem.* **2019**, *11*, 872.

[9] M. Brookhart, B. Grant, A. F. Volpe, Jr., « [(3,5-(CF3)2C6H3)4B]-[H(OEt2)2]+: a convenient reagent for generation and stabilization of cationic, highly electrophilic organometallic complexes » *Organometallics* **1992**, *11*, 3920.

[10] J. W. McDonald, G. D. Friesen, L. D. Rosenhein, W. E. Newton, « Syntheses and characterization of ammonium and tetraalkylammonium thiomolybdates and thiotungstates » *Inorganica Chim. Acta* **1983**, *72*, 205.

[11] S. Kobayashi, I. Hachiya, « Lanthanide Triflates as Water-Tolerant Lewis Acids. Activation of Commercial Formaldehyde Solution and Use in the Aldol Reaction of Silyl Enol Ethers with Aldehydes in Aqueous Media » *J. Org. Chem.* **1994**, *59*, 3590.

[12] Y.-Z. Pan, Q.-Y. Hua, L.-S. Lin, Y.-B. Qiu, J.-L. Liu, A.-J. Zhou, W.-Q. Lin, J.-D. Leng, « A slowly magnetic relaxing Sm(III) monomer with a D5h equatorial compressed ligand field » *Inorg. Chem. Front.* **2020**, *7*, 2335.

[13] S. Yu, Z. Chen, H. Hu, B. Li, Y. Liang, D. Liu, H. Zou, D. Yao, F. Liang, « Two mononuclear dysprosium(III) complexes with their slow magnetic relaxation behaviors tuned by coordination geometry » *Dalton Trans.* **2019**, *48*, 16679.

[14] B.-Y. Chen, M.-Y. Tsai, Y.-C. Su, P.-H. Lin, J. Long, « Synthesis, structures and magnetic properties of dysprosium(III) complexes based on amino-bis(benzotriazole phenolate) and nitrophenolates: influence over the slow relaxation of the magnetization » *CrystEngComm* **2021**, *23*, 8343.

[15] B. Ali, X.-L. Li, F. Gendron, B. Le Guennic, J. Tang, « A new class of DyIII-SIMs associated with a guanidine-based ligand » *Dalton Trans.* **2021**, *50*, 5146.

[16] A. K. Bar, P. Kalita, J.-P. Sutter, V. Chandrasekhar, « Pentagonal-Bipyramid Ln(III) Complexes Exhibiting Single-Ion-Magnet Behavior: A Rational Synthetic Approach for a Rigid Equatorial Plane » *Inorg. Chem.* **2018**, *57*, 2398.

[17] X.-C. Huang, Q.-C. Huang, H.-Y. Mu, S.-C. Yan, Z.-J. Han, D. Shao, X.-R. Chen, J. Dong, J.-J. Kong, Y.-Q. Zhang, « Two mononuclear chromophore coumarin-based Dy(III) complexes with magnetic relaxation and luminescent properties » *Journal of Molecular Structure* **2024**, *1302*, 137408.

[18] I. Fdez. Galván, M. Vacher, A. Alavi, C. Angeli, F. Aquilante, J. Autschbach, J. J. Bao, S. I. Bokarev, N. A. Bogdanov, R. K. Carlson, L. F. Chibotaru, J. Creutzberg, N. Dattani, M. G. Delcey, S. S. Dong, A. Dreuw, L. Freitag, L. M. Frutos, L. Gagliardi, F. Gendron, A. Giussani, L. González, G. Grell, M. Guo, C. E. Hoyer, M. Johansson, S. Keller, S. Knecht, G. Kovačević, E. Källman, G. Li Manni, M. Lundberg, Y. Ma, S. Mai, J. P. Malhado, P. Å. Malmqvist, P. Marquetand, S. A. Mewes, J. Norell, M. Olivucci, M. Oppel, Q. M. Phung, K. Pierloot, F. Plasser, M. Reiher, A. M. Sand, I. Schapiro, P. Sharma, C. J. Stein, L. K. Sørensen, D. G. Truhlar, M. Ugandi, L. Ungur, A. Valentini, S. Vancoillie, V. Veryazov, O. Weser, T. A. Wesołowski, P.-O. Widmark, S. Wouters, A. Zech, J. P. Zobel, R. Lindh, « OpenMolcas: From Source Code to Insight » *Journal of Chemical Theory and Computation* **2019**, *15*, 5925.

[19] G. Li Manni, I. Fdez. Galván, A. Alavi, F. Aleotti, F. Aquilante, J. Autschbach, D. Avagliano, A. Baiardi, J. J. Bao, S. Battaglia, L. Birnoschi, A. Blanco-González, S. I. Bokarev, R. Broer, R. Cacciari, P. B. Calio, R. K. Carlson, R. Carvalho Couto, L. Cerdán, L. F. Chibotaru, N. F. Chilton, J. R. Church, I. Conti, S. Coriani, J. Cuéllar-Zuquin, R. E. Daoud, N. Dattani, P. Decleva, C. de Graaf, M. G. Delcey, L. De Vico, W. Dobrautz, S. S. Dong, R. Feng, N. Ferré, M. Filatov, L. Gagliardi, M. Garavelli, L. González, Y. Guan, M. Guo, M. R. Hennefarth, M. R. Hermes, C. E. Hoyer, M. Huix-Rotllant, V. K. Jaiswal, A. Kaiser, D. S. Kaliakin, M. Khamesian, D. S. King, V. Kochetov, M. Krośnicki, A. A. Kumaar, E. D. Larsson, S. Lehtola, M.-B. Lepetit, H. Lischka, P. López Ríos, M. Lundberg, D. Ma, S. Mai, P. Marquetand, I. C. D. Merritt, F. Montorsi, M. Mörchen, A. Nenov, V. H. A. Nguyen, Y. Nishimoto, M. S. Oakley, M. Olivucci, M. Oppel, D. Padula, R. Pandharkar, Q. M. Phung, F. Plasser, G. Raggi, E. Rebolini, M. Reiher, I. Rivalta, D. Roca-Sanjuán, T. Romig, A. A. Safari, A. Sánchez-Mansilla, A. M. Sand, I. Schapiro, T. R. Scott, J. Segarra-Martí, F. Segatta, D.-C. Sergentu, P. Sharma, R. Shepard, Y. Shu, J. K. Staab, T. P. Straatsma, L. K. Sørensen, B. N. C. Tenorio, D. G. Truhlar, L. Ungur, M. Vacher, V. Veryazov, et al., « The OpenMolcas Web: A Community-Driven Approach to Advancing Computational Chemistry » *Journal of Chemical Theory and Computation* **2023**, *19*, 6933.

[20] M. Douglas, N. M. Kroll, « Quantum electrodynamical corrections to the fine structure of helium » *Ann. Phys.* **1974**, *82*, 89.

[21] B. A. Hess, « Relativistic electronic-structure calculations employing a two-component no-pair formalism with external-field projection operators » *Phys. Rev. A.* **1986**, *33*, 3742.

[22] A. Wolf, M. Reiher, B. A. Hess, « The generalized Douglas–Kroll transformation » *The Journal of Chemical Physics* **2002**, *117*, 9215.

[23] B. O. Roos, P. R. Taylor, P. E. M. Sigbahn, « A complete active space SCF method (CASSCF) using a density matrix formulated super-CI approach » *Chem. Phys.* **1980**, *48*, 157.

[24] K. Andersson, P. A. Malmqvist, B. O. Roos, A. J. Sadlej, K. Wolinski, « Second-order perturbation theory with a CASSCF reference function » *J. Phys. Chem.* **1990**, *94*, 5483.

[25] P. Å. Malmqvist, B. O. Roos, B. Schimmelpfennig, « The restricted active space (RAS) state interaction approach with spin–orbit coupling » *Chem. Phys. Lett.* **2002**, *357*, 230.

[26] P.-Å. Malmqvist, B. O. Roos, « The CASSCF state interaction method » *Chem. Phys. Lett.* **1989**, *155*, 189.

[27] B. A. Heß, C. M. Marian, U. Wahlgren, O. Gropen, « A mean-field spin-orbit method applicable to correlated wavefunctions » *Chem. Phys. Lett.* **1996**, *251*, 365.

[28] L. F. Chibotaru, L. Ungur, « Ab initio calculation of anisotropic magnetic properties of complexes. I. Unique definition of pseudospin Hamiltonians and their derivation » *The Journal of Chemical Physics* **2012**, *137*.

[29] L. F. Chibotaru, L. Ungur, A. Soncini, « The Origin of Nonmagnetic Kramers Doublets in the Ground State of Dysprosium Triangles: Evidence for a Toroidal Magnetic Moment » *Angew. Chem. Int. Ed.* **2008**, *47*, 4126.

[30] F. Aquilante, P.-Å. Malmqvist, T. B. Pedersen, A. Ghosh, B. O. Roos, « Cholesky Decomposition-Based Multiconfiguration Second-Order Perturbation Theory (CD-CASPT2): Application to the Spin-State Energetics of CoIII(diiminato)(NPh) » *Journal of Chemical Theory and Computation* **2008**, *4*, 694.

[31] P.-O. Widmark, P.-Å. Malmqvist, B. O. Roos, « Density matrix averaged atomic natural orbital (ANO) basis sets for correlated molecular wave functions » *Theor. Chim. Acta* **1990**, *77*, 291.

[32] B. O. Roos, R. Lindh, P.-Å. Malmqvist, V. Veryazov, P.-O. Widmark, « Main Group Atoms and Dimers Studied with a New Relativistic ANO Basis Set » *J. Phys. Chem. A* **2004**, *108*, 2851.

[33] B. O. Roos, R. Lindh, P.-Å. Malmqvist, V. Veryazov, P.-O. Widmark, A. C. Borin, « New Relativistic Atomic Natural Orbital Basis Sets for Lanthanide Atoms with Applications to the Ce Diatom and LuF3 » *J. Phys. Chem. A* **2008**, *112*, 11431.

[34] M. F. Rüger, R. , T. Trnka, A. Yakovlev, E. van Lenthe, P. Philipsen, T. van Vuren, B. Klumpers, T. Soini, **2022**.

[35] E. J. Baerends, T. Ziegler, A. J. Atkins, J. Autschbach, O. Baseggio, D. Bashford, **2022**.

[36] E. v. Lenthe, E. J. Baerends, J. G. Snijders, « Relativistic regular two-component Hamiltonians » *The Journal of Chemical Physics* **1993**, *99*, 4597.

[37] E. van Lenthe, A. Ehlers, E.-J. Baerends, « Geometry optimizations in the zero order regular approximation for relativistic effects » *The Journal of Chemical Physics* **1999**, *110*, 8943.

[38] J. P. Perdew, K. Burke, M. Ernzerhof, « Generalized Gradient Approximation Made Simple [Phys. Rev. Lett. 77, 3865 (1996)] » *Phys. Rev. Lett.* **1997**, *78*, 1396.

[39] Y. Zhang, W. Yang, « Comment on ``Generalized Gradient Approximation Made Simple'' » *Phys. Rev. Lett.* **1998**, *80*, 890.

[40] E. Van Lenthe, E. J. Baerends, « Optimized Slater-type basis sets for the elements 1–118 » *J. Comput. Chem.* **2003**, *24*, 1142.

[41] L. Gagliardi, R. Lindh, G. Karlström, « Local properties of quantum chemical systems: The LoProp approach » *The Journal of Chemical Physics* **2004**, *121*, 4494.

[42] K. Zhang, V. Montigaud, O. Cador, G.-P. Li, B. Le Guennic, J.-K. Tang, Y.-Y. Wang, « Tuning the Magnetic Interactions in Dy(III)4 Single-Molecule Magnets » *Inorg. Chem.* **2018**, *57*, 8550.

[43] G. Huang, G. Fernandez-Garcia, I. Badiane, M. Camarra, S. Freslon, O. Guillou, C. Daiguebonne, F. Totti, O. Cador, T. Guizouarn, B. Le Guennic, K. Bernot, « Magnetic Slow Relaxation in a Metal–Organic Framework Made of Chains of Ferromagnetically Coupled Single-Molecule Magnets » *Chem. Eur. J.* **2018**, *24*, 6983.

[44] M. D. Korzyński, Z. J. Berkson, B. Le Guennic, O. Cador, C. Copéret, « Leveraging Surface Siloxide Electronics to Enhance the Relaxation Properties of a Single-Molecule Magnet » *J. Am. Chem. Soc.* **2021**, *143*, 5438.

[45] F. Neese, « Software update: The ORCA program system—Version 5.0 » *WIREs Computational Molecular Science* **2022**, *12*, e1606.

[46] F. Neese, « The SHARK integral generation and digestion system » *J. Comput. Chem.* **2023**, *44*, 381.

[47] D. Bykov, P. Taras, I. Róbert, K. Simone, B. Ute, V. Edward, F. and Neese, « Efficient implementation of the analytic second derivatives of Hartree–Fock and hybrid DFT energies: a detailed analysis of different approximations » *Molecular Physics* **2015**, *113*, 1961.

[48] J. P. Perdew, K. Burke, M. Ernzerhof, « Generalized Gradient Approximation Made Simple » *Phys. Rev. Lett.* **1996**, *77*, 3865.

[49] J. D. Rolfes, F. Neese, D. A. Pantazis, « All-electron scalar relativistic basis sets for the elements Rb–Xe » *J. Comput. Chem.* **2020**, *41*, 1842.

[50] S. Grimme, J. Antony, S. Ehrlich, H. Krieg, « A consistent and accurate ab initio parametrization of density functional dispersion correction (DFT-D) for the 94 elements H-Pu » *The Journal of Chemical Physics* **2010**, *132*.

[51] S. Grimme, S. Ehrlich, L. Goerigk, « Effect of the damping function in dispersion corrected density functional theory » *J. Comput. Chem.* **2011**, *32*, 1456.

[52] F. Neese, « An improvement of the resolution of the identity approximation for the formation of the Coulomb matrix » *J. Comput. Chem.* **2003**, *24*, 1740.
